# Interactions among acute respiratory viruses in Beijing, Chongqing, Guangzhou, and Shanghai, China, 2009–2019

**DOI:** 10.1111/irv.13212

**Published:** 2023-11-12

**Authors:** Zachary J. Madewell, Li‐Ping Wang, Natalie E. Dean, Hai‐Yang Zhang, Yi‐Fei Wang, Xiao‐Ai Zhang, Wei Liu, Wei‐Zhong Yang, Ira M. Longini, George F. Gao, Zhong‐Jie Li, Li‐Qun Fang, Yang Yang, The Chinese Centers for Disease Control and Prevention (CDC) Etiology of Respiratory Infection Surveillance Study Team, Wei‐Zhong Yang, George F. Gao, Zhong‐Jie Li, Li‐Ping Wang, Xiang Ren, Yi‐Fei Wang, Meng‐Jie Geng, Xin Wang, Huai‐Qi Jing, Wen‐Bo Xu, Ai‐Li Cui, Yu‐Juan Shen, Yan‐Yan Jiang, Qiao Sun, Li‐Peng Hao, Chu‐Chu Ye, Wei Liu, Liu‐Yu Huang, Yong Wang, Wen‐Yi Zhang, Ying‐Le Liu, Jian‐Guo Wu, Qi Zhang, Wei‐Yong Liu, Zi‐Yong Sun, Fa‐Xian Zhan, Ying Xiong, Lei Meng, De‐Shan Yu, Sheng‐Cang Zhao, Wen‐Rui Wang, Xia Lei, Juan‐Sheng Li, Yan Zhang, Yan‐Bo Wang, Fu‐Cai Quan, Zhi‐Jun Xiong, Li‐Ping Liang, Quan‐E Chang, Yun Wang, Ping Wang, Zuo‐Sen Yang, Ling‐Ling Mao, Jia‐Meng Li, Li‐Kun Lv, Jun Xu, Chang Shu, Xiao Chen, Yu Chen, Yan‐Jun Zhang, Lun‐Biao Cui, Kui‐Cheng Zheng, Xing‐Guo Zhang, Xi Zhang, Li‐Hong Tu, Zhi‐Gang Yi, Wei Wang, Shi‐Wen Zhao, Xiao‐Fang Zhou, Xiao‐Fang Pei, Tian‐Li Zheng, Xiao‐Ni Zhong, Qin Li, Hua Ling, Shi‐Jun Li, Shu‐Sen He, Meng‐Feng Li, Jun Li, Xun Zhu, Chang‐Wen Ke, Hong Xiao, Biao Di, Ying Zhang, Hong‐Wei Zhou, Nan Yu, Hong‐Jian Li, Fang Yang, Fu‐Xiang Wang, Jun Wang

**Affiliations:** ^1^ Department of Biostatistics, College of Public Health and Health Professions & Emerging Pathogens Institute University of Florida Gainesville Florida USA; ^2^ Division of Infectious Disease Key Laboratory of Surveillance and Early‐Warning on Infectious Diseases, Chinese Center for Disease Control and Prevention Beijing China; ^3^ Department of Biostatistics and Bioinformatics Emory University Atlanta Georgia USA; ^4^ State Key Laboratory of Pathogen and Biosecurity Beijing Institute of Microbiology and Epidemiology Beijing China; ^5^ Department of Statistics, Franklin College of Arts and Sciences University of Georgia Athens Georgia USA

**Keywords:** acute respiratory infections, co‐infection, pathogen interactions, viral interference, viral synergism

## Abstract

**Background:**

A viral infection can modify the risk to subsequent viral infections via cross‐protective immunity, increased immunopathology, or disease‐driven behavioral change. There is limited understanding of virus–virus interactions due to lack of long‐term population‐level data.

**Methods:**

Our study leverages passive surveillance data of 10 human acute respiratory viruses from Beijing, Chongqing, Guangzhou, and Shanghai collected during 2009 to 2019: influenza A and B viruses; respiratory syncytial virus A and B; human parainfluenza virus (HPIV), adenovirus, metapneumovirus (HMPV), coronavirus, bocavirus (HBoV), and rhinovirus (HRV). We used a multivariate Bayesian hierarchical model to evaluate correlations in monthly prevalence of test‐positive samples between virus pairs, adjusting for potential confounders.

**Results:**

Of 101,643 lab‐tested patients, 33,650 tested positive for any acute respiratory virus, and 4,113 were co‐infected with multiple viruses. After adjusting for intrinsic seasonality, long‐term trends and multiple comparisons, Bayesian multivariate modeling found positive correlations for HPIV/HRV in all cities and for HBoV/HRV and HBoV/HMPV in three cities. Models restricted to children further revealed statistically significant associations for another ten pairs in three of the four cities. In contrast, no consistent correlation across cities was found among adults. Most virus–virus interactions exhibited substantial spatial heterogeneity.

**Conclusions:**

There was strong evidence for interactions among common respiratory viruses in highly populated urban settings. Consistent positive interactions across multiple cities were observed in viruses known to typically infect children. Future intervention programs such as development of combination vaccines may consider spatially consistent virus–virus interactions for more effective control.

## 1 INTRODUCTION

Infections caused by one virus can potentially modify the probability of other viral infections via different causal pathways, as documented in the clinical setting.^1^ Mechanisms for such interferences range from cellular‐level interactions (e.g., cross‐reactive or innate immune responses, cell damage), to host‐level interactions (tissue damage), and to population‐level interactions (behavioral responses to disease such as self‐isolation).^2^, ^3^ Viral infections may interfere with subsequent viral infections by competing for host resources or providing long‐term specific or transient nonspecific immunity following the initial infection, particularly among taxonomically similar viruses.^4^, ^5^ There is evidence that influenza types and subtypes interact with each other via cross‐immunity or genetic changes/exchanges,^2^ for example, HA‐stalk‐specific neutralizing antibodies cross‐react to both influenza A virus (IAV) and influenza B virus (IBV).^6^ Interferences may also occur between genetically or antigenically distant viral species. At the host level, human rhinovirus (HRV) infections may protect against subsequent IAV infections via antiviral defenses triggered in the airway mucosa.^7^ One study found that infection with IAV can increase expression of ACE2 receptors and thus affect cell susceptibility to SARS‐CoV‐2.^8^ At the population level, viral interference may be induced when an epidemic caused by one virus hastens or delays an epidemic caused by another virus.^9^ For example, nonpharmaceutical interventions implemented to control SARS‐CoV‐2 were associated with substantial changes in the circulation of other viruses.^10^

To understand transmission patterns, risk drivers and needed controls of a specific pathogen and to plan an appropriate public health response, it is necessary to understand how that pathogen interacts with other co‐circulating pathogens. For example, public health interventions such as vaccination campaigns targeting a particular virus may impact the abundance of other viruses competing for or occupying the same niche.^9^ The increasing availability of population‐level etiological surveillance data in recent decades has greatly facilitated epidemiological studies of interactions between co‐circulating pathogens. For instance, data from a virological surveillance system in the Netherlands indicated that early seasonal epidemics of IAV may affect subsequent incidences of respiratory syncytial virus (RSV) and human coronavirus (HCoV).^11^ It is often difficult, however, to determine whether population‐level interferences are true biological virus–virus interactions or reflections of changes in healthcare‐seeking behavior (e.g., lockdown) or surveillance practices (altered laboratory testing frequency or techniques). The difficulty partly lies in the fact that, in routine healthcare settings, patients are often tested for only one or two pathogens based on clinical symptoms and/or local surveillance data about circulating pathogens. Therefore, incidence rates are not directly comparable between pathogens as the samples may come from very different subpopulations. This problem can be alleviated by testing a comprehensive panel of pathogens on the same representative clinical samples; yet adequate statistical power to detect interactions among multiple pathogens requires a relatively large sample size covering a relatively long timespan, which is not available in most studies.

Statistical methods are needed to better tease out potential biological interactions between viruses in the presence of confounding socioenvironmental factors associated with virus survival and transmission, such as climatic conditions, age‐dependent contact patterns, and changes in testing frequencies. For example, shared seasonality patterns between influenza and RSV often confound interpretation of temporal correlation between the two viruses. One study in Scotland accounted for these factors via hierarchical autoregressive modeling to examine interactions between multiple groups of respiratory viruses over several years.^12^, ^13^ The analysis identified several significant positive (human metapneumovirus [HMPV] and RSV; human parainfluenza viruses [HPIV] 1 and 2) and negative (IAV and HRV; IBV and human adenovirus [HAdV]) interactions.

Herein, we adapt a Bayesian virus–virus interaction framework designed for surveillance data with relatively dense sampling^13^ to the situation with frequent sparse sampling and zero incidence. This adapted framework handles both dense and sparse sampling as well as zero inflation and is hence more general. In addition, we remove the impact of intrinsic seasonality and long‐term trend of each virus on the evaluation of virus–virus interaction, thus providing more robust statistical evidence of virus–virus interactions. We apply this model to passive surveillance data from four major cities in China over an 11‐year period to evaluate interactions between 10 respiratory viruses for which most patients were tested: IAV, IBV, RSV‐A, RSV‐B, HPIV, HAdV, HMPV, HCoV, human bocavirus (HBoV), and HRV.

## 2 METHODS

### 2.1 Study population

Our data on acute respiratory infections (ARIs) were collected by the Surveillance for Etiology of Respiratory Infections program, an ongoing nationwide passive surveillance program initiated from 2009, which covers 314 sentinel hospitals in all 31 provinces in China. The ARIs were defined as (1) at least one of the following conditions: fever, abnormal white blood cell differentials, leukocytosis or leukopenia; and (2) at least one of the following symptoms/signs: cough, chills, expectoration, nasal congestion, sore throat, chest pain, tachypnea, and abnormal pulmonary breath sounds. In compliance with the standard operating protocol of surveillance developed by China CDC, a random sample of inpatients and outpatients presenting ARIs were enrolled, and nasopharyngeal specimens were collected from enrolled patients prior to any treatment to be tested for the presence of influenza virus (types A, B and C), RSV‐A and RSV‐B, HPIV types 1–4, HAdV, HMPV, HCoV, HBoV, and HRV. All viruses were tested by reverse transcription‐polymerase chain reaction (RT‐PCR) except HAdV and HBoV, which were tested by PCR. Details about the laboratory procedures are given elsewhere.^14^

We focus on four metro cities that consistently tested patients for these viruses throughout the 11‐year period: Beijing, Chongqing, Guangzhou, and Shanghai. Nearly all enrolled patients presenting with acute respiratory symptoms were tested for all respiratory viruses during 2009–2019. There was a lower testing rate, however, for HRV during 2009–2011, so we restricted analyses related to HRV to 2012–2019. We also excluded influenza C from analysis and grouped HPIV 1–4 viruses into a single HPIV group due to the relatively low frequency of each individual virus. Analyses were done for the total population studied and separately for children <18 years and adults ≥18 years. There was a lower testing rate for adults in Chongqing in 2011 and 2015–2019; therefore, that subgroup was excluded from age‐stratified analyses.

### 2.2 Unadjusted correlation

For correlation analyses, we aggregated data into monthly infection counts for each virus to evaluate the population‐level covariation patterns between viruses in each city (132 total months). We calculated a measure of prevalence of positive tests for each virus as the number of infected patients over the total number of patients tested for each virus for each month. We calculated weighted Pearson's coefficients and 95% confidence intervals (CIs) to evaluate unadjusted correlations in monthly infection prevalence between each of the 45 virus pairs with statistical significance set at *p* < 0.05 and *p* < 0.10, with weights corresponding to the number of tests administered. The *p* values were corrected for multiple comparisons using the Benjamin–Hochberg procedure,^15^ and false discovery rate (FDR)‐adjusted *q* values were reported. Statistical significance was defined as *q* ≤ 0.10, that is, controlling the FDR at 10%. We also calculated weighted Pearson's coefficients in monthly infection prevalence by sex for each virus in each city. At the individual level, we also calculated the odds of co‐infection for each virus with each of the other viruses (45 virus pairs) in each city and used Fisher's exact test for statistical significance, and similarly, FDR‐adjusted *q* values were reported.

### 2.3 Multivariate Bayesian hierarchical model

Simple correlations among respiratory viruses may be confounded by changes in testing frequencies, temporal autocorrelation, and other factors. To address these challenges, we used a multivariate Bayesian hierarchical model, adapting an approach initially designed by Mair et al.^13^ (for detailed procedures, see Supporting information).

Before applying the hierarchical model, we smoothed observed monthly infection counts for each virus using logistic regression, adjusting for common risk factors for infection. We fitted generalized linear models for each virus and city, incorporating harmonic functions to account for seasonality and polynomials of year to capture long‐term trends, while also adjusting for sex and age groups.

In the second stage, we modeled interactions between pairs of viruses separately. We introduced a multiplicative random odds ratio (OR) to describe how the realized prevalence of positive samples for each virus deviates from the expected prevalence. This random OR is related to the random effect φ, which follows a multivariate AR (1) structure to capture correlations between viruses. Specifically, we used the correlation coefficient *ρ* to characterize virus–virus interactions. A range of (−3, 3) for *ρ* effectively covers a range of (−0.95, 0.95) for the correlation coefficient. A fundamental distinction in our model compared with previous work^13^ is the use of a zero‐inflated binomial model for the monthly number of positive tests to account for excess zeros and the variability in the number of tests conducted each month.

We used 10 chains, each with a burn‐in of 20,000 iterations, followed by 600 thinned draws from 60,000 additional iterations per chain, ensuring convergence (Figure S1). Models were fitted using the package R2jags in R statistical software (R Development Core Team, Vienna, Austria).^16^

## 3 RESULTS

### 3.1 Study population

Our dataset included 216,030 patients from January 2009 to December 2019, of whom 115,622 (53.5%) were from Beijing, Chongqing, Guangzhou, and Shanghai, four large metro cities each with a population size of 15–31 million. Of 101,643 patients from the four cities who were tested for any of the viruses of interest, 44.7% were from Beijing, 59.2% were male, and 50.8% were children (Table 1). Of those patients, 63,202 (62.2%) were tested for all 10 viruses and 28,212 (27.8%) patients were tested for nine viruses (Table S1). Of patients who were tested for nine viruses, 28 014 (99.3%) were not tested for HRV. Unless stated otherwise, all subsequent analyses were based on the 101,643 patients from the four cities.

**TABLE 1:** Descriptive statistics of patients who were tested for any of 10 respiratory viruses in four metro cities (Beijing, Shanghai, Chongqing, and Guangzhou) of China, 2009–2019.

Characteristics	Number of patients	
	*n* = 101,643	%
Sex		
Female	41,518	40.8
Male	60,125	59.2
Age group (in years)		
<5	34,571	34.0
5–18	17,063	16.8
18–40	25,344	24.9
≥40	24,665	24.3
City		
Beijing	45,433	44.7
Shanghai	23,465	23.1
Guangzhou	22,875	22.5
Chongqing	9870	9.7
Tested for influenza A virus (IAV)	101,466	99.8
Positive	10,901	10.7
Negative	90,565	89.3
Tested for influenza B virus (IBV)	101,466	99.8
Positive	3194	3.1
Negative	98,272	96.9
Tested for respiratory syncytial virus A (RSV‐A)	96,140	94.6
Positive	3103	3.2
Negative	93,037	96.8
Tested for respiratory syncytial virus B (RSV‐B)	96,140	94.6
Positive	2462	2.6
Negative	93,678	97.4
Tested for human parainfluenza virus (HPIV)	96,186	94.6
Positive	5034	5.2
Negative	91,152	94.8
Tested for human adenovirus (HAdV)	96,285	94.7
Positive	3609	3.7
Negative	92,676	96.3
Tested for human metapneumovirus (HMPV)	95,862	94.3
Positive	1691	1.8
Negative	94,171	98.2
Tested for human coronavirus (HCoV)	95,665	94.1
Positive	2079	2.2
Negative	93,586	97.8
Tested for human bocavirus (HBoV)	91,512	90.0
Positive	1782	1.9
Negative	89,730	98.1
Tested for human rhinovirus (HRV)	63,594	62.6
Positive	4549	7.2
Negative	59,045	92.8

### 3.2 Overall prevalence and co‐infection rates

**FIGURE 1:**
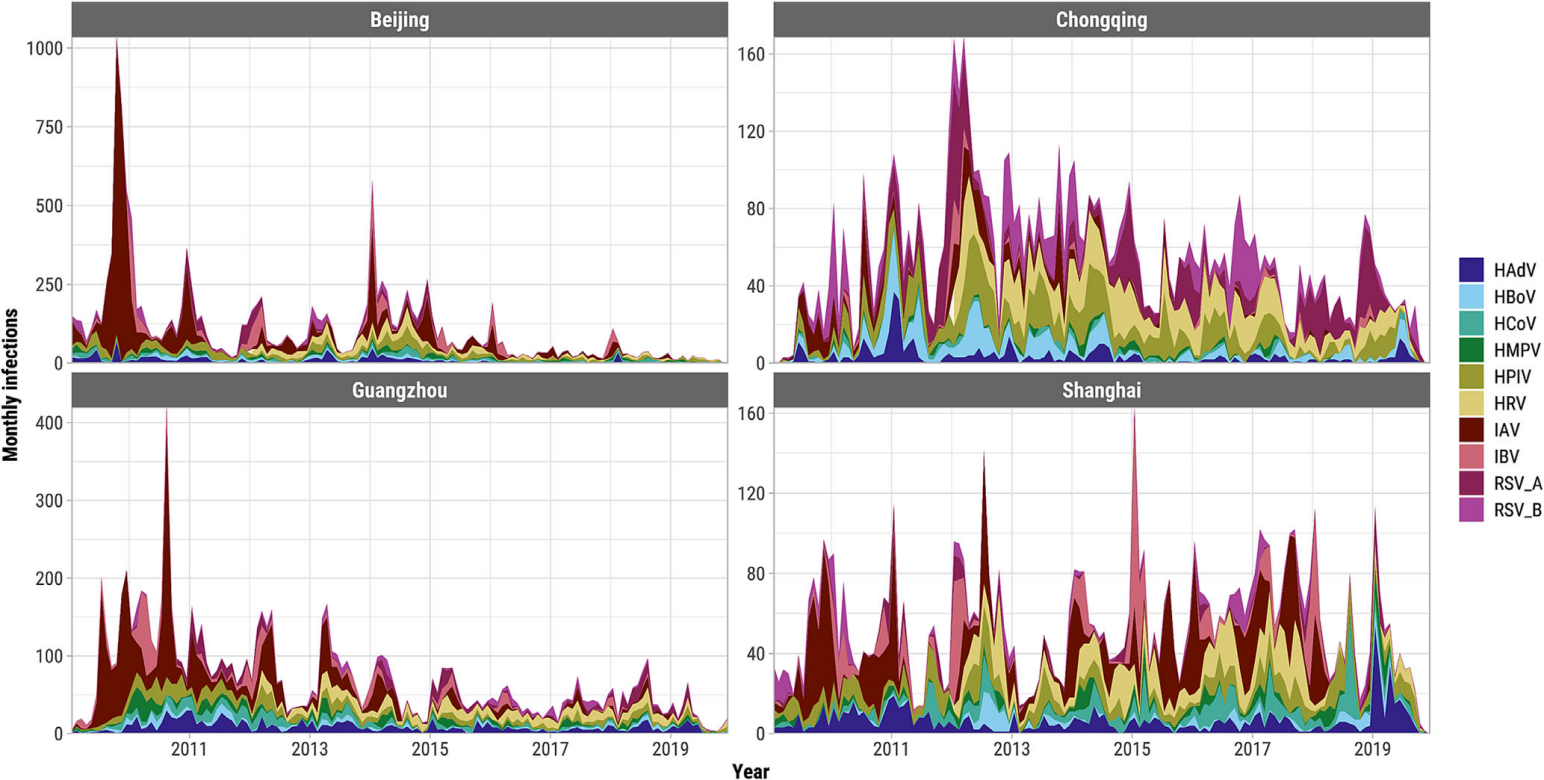
Laboratory‐detected respiratory viral infections for each month from January 2009 to December 2019 in four metro cities, China.

**FIGURE 2:**
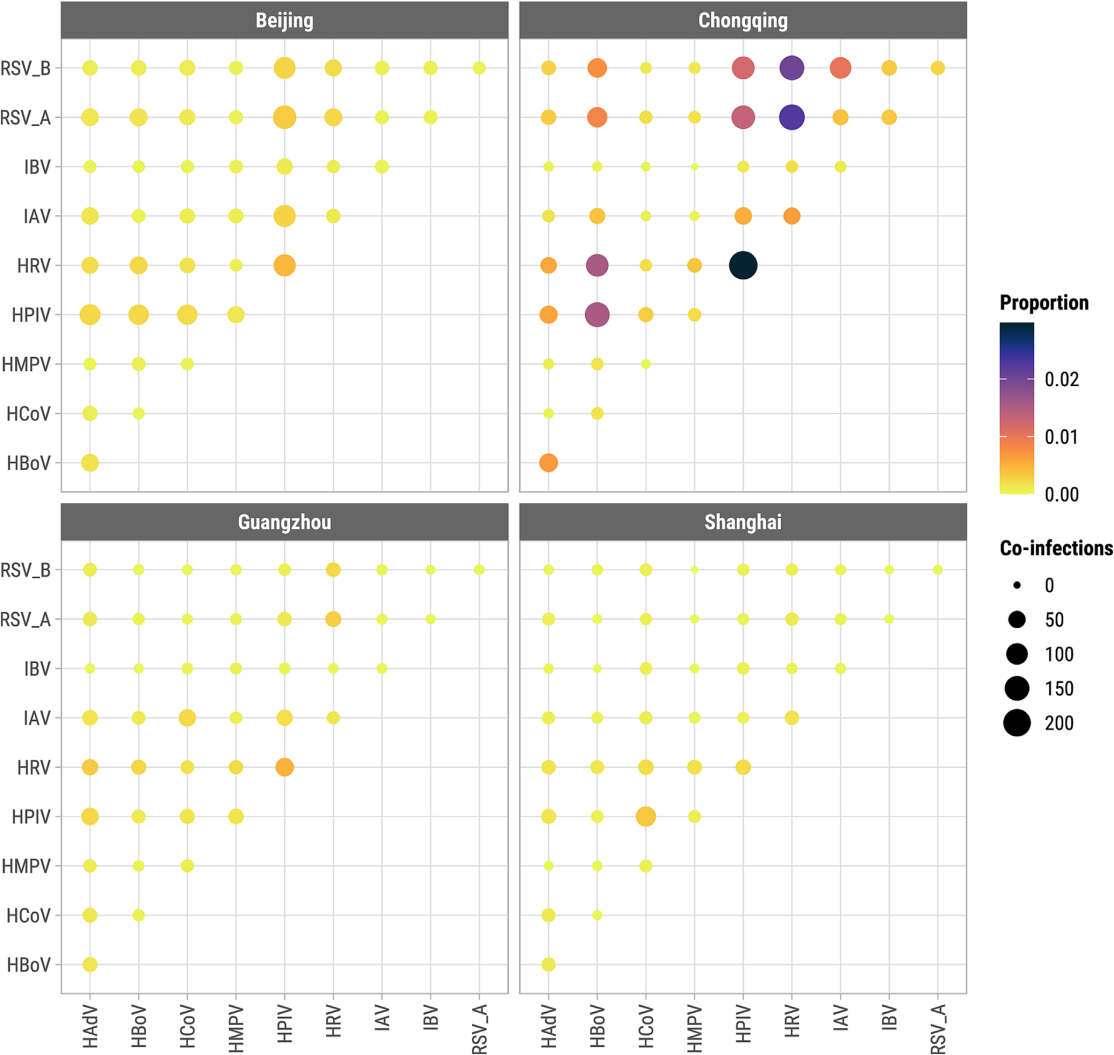
Frequency of confirmed co‐infections for each pair of respiratory viruses shown by size and proportion of each co‐infection pair among all tested samples shown by color from January 2009 to December 2019 in four metro cities, China.

There were 33,650/101,643 (33.1%) patients who tested positive for any of the 10 viruses studied (Figure S2), of whom 4113 (12.2%) were co‐infected with more than one acute respiratory virus (Figure S3). The overall proportion of co‐infection among test‐positive samples was highest in Chongqing (15.1%) and lowest in Shanghai (2.3%). There was substantial variation in monthly prevalence of patients testing positive (test‐positive rate) for any acute respiratory virus in each city, and the average test‐positive rate was much higher in Chongqing but similar in the other three cities (Figure S3). Monthly prevalences were significantly correlated between the two sexes in each city for each virus, and the correlations tended to be higher for influenza viruses and RSVs than for other viruses (Figure S4). The most frequent viral infections were IAV (28.4% of infections on average across all cities and years), HPIV (13.1%), and HRV (11.8%) (Figure 1). IAV/H1N1 (mainly A/H1N1 pdm09) dominated infections detected in 2009–2010 with additional waves through 2011, many of which were detected in Beijing (Figure 1). IAV was also the most prevalent acute respiratory virus from 2009 to 2019 in Beijing (12.9% of patients tested positive), Guangzhou (11.9%), and Shanghai (7.5%) (Figure S5). The majority of patients tested in Chongqing were children and the most prevalent viruses were HRV (20.9%) and HPIV (16.7%) (Figures S5 and S6). The relative prevalence of the acute respiratory viruses fluctuated month‐to‐month with influenza viruses and RSV showing the strongest seasonality (Figure S7). The highest proportion of co‐infections among all tested samples were HPIV/HRV in Beijing (0.5% of all tested samples), Chongqing (3.0%), and Guangzhou (0.5%), and HPIV/HCoV in Shanghai (0.4%) (Figure 2).

At the individual level, we evaluated for each pair of viruses the OR of co‐infection by city but lumping data from all 11 years, while controlling for FDR ≤ 0.10. Of 45 pairwise tests in each city, 36 were significant at *q* ≤ 0.10 in Beijing (24 positive [ORs > 1], 12 negative [ORs< 1]), 30 in Shanghai (10 positive, 20 negative), 26 in Guangzhou (4 positive, 22 negative), and 22 in Chongqing (10 positive, 12 negative) (Figure S8). In Beijing, all 24 significant non‐influenza interactions were positive. The highest odds of co‐infection in each city were between RSV‐A and HBoV in Beijing (OR = 5.6, *q* < 0.001), HPIV and HCoV in Shanghai (OR = 3.9, *q* < 0.001), HRV and HBoV in Guangzhou (OR = 2.6, *q* < 0.001), and IBV and RSV‐B in Chongqing (OR = 2.2, *q* < 0.001).

### 3.3 Unadjusted correlations between viruses

**FIGURE 3:**
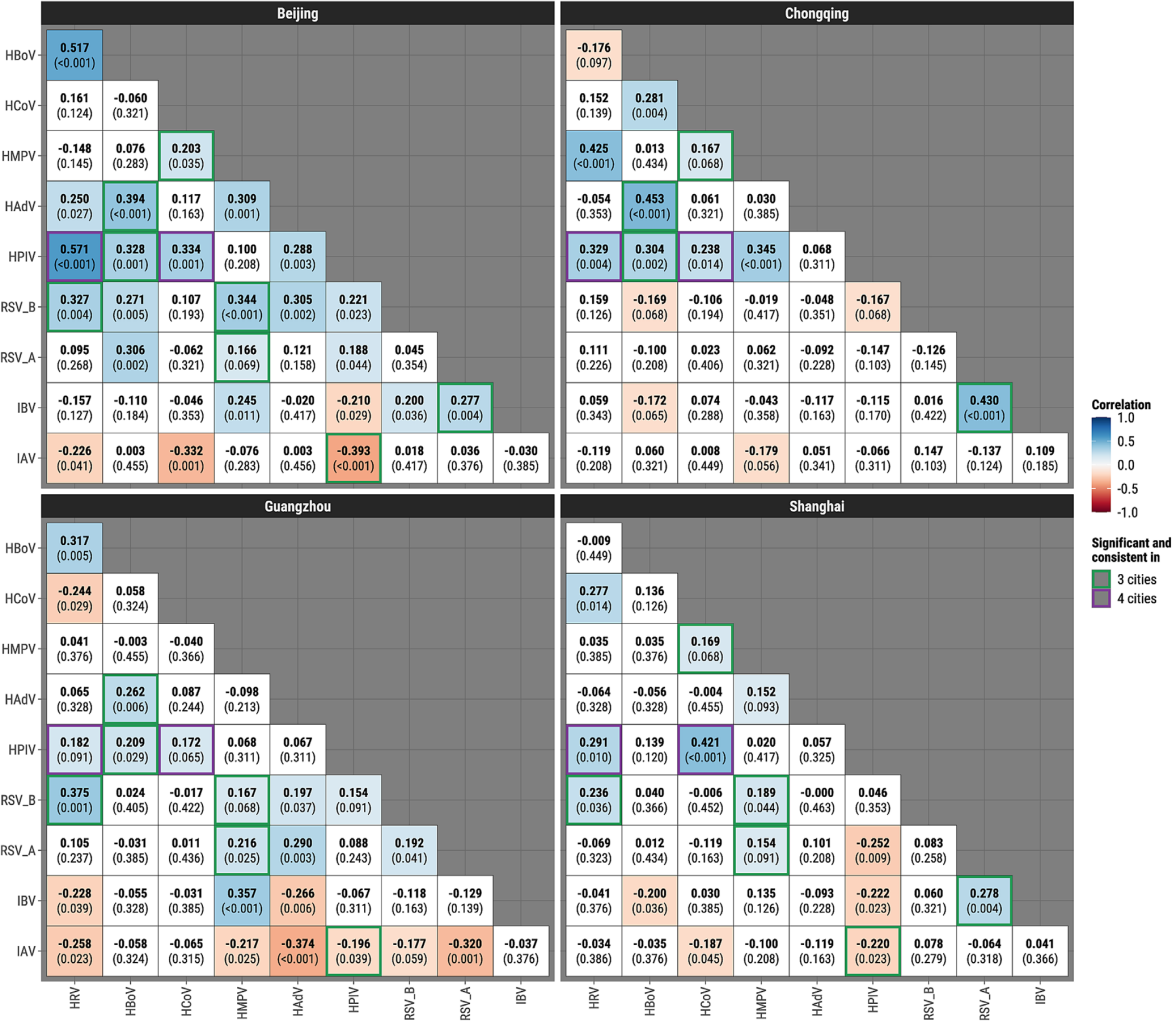
Weighted Pearson's correlation coefficients for the total population studied and *q*‐values of monthly prevalence for each pair of respiratory viruses in four metro cities of China, where weights are the numbers of tests administered, China. The *q*‐values represent statistical evidence adjusted for multiple comparisons by controlling the false discovery rate. Significant correlations (*q* ≤ 0.10) are shown in color. Blue and red indicate positive and negative coefficients, respectively. Green and purple borders indicate virus pairs statistically significant with consistent directions in three and four cities, respectively.

We then assessed weighted Pearson's correlations between monthly prevalence of virus pairs for each city. When controlling for FDR ≤ 0.10 (Figure 3), there were 24 significant virus–virus correlations in Beijing (20 positive, four negative), 22 in Guangzhou (13 positive, nine negative), 14 in Chongqing (nine positive, five negative), and 14 in Shanghai (nine positive, five negative). HPIV/HCoV (0.17 to 0.42) and HPIV/HRV (0.18 to 0.57) were the only pairs with statistically significant correlations in a direction consistent across all four cities. Pairs that were consistent in three cities include IAV/HPIV (−0.39 to −0.20), IBV/RSV‐A (0.28 to 0.43), RSV‐A/HMPV (0.15 to 0.22), RSV‐B/HMPV (0.17 to 0.34), RSV‐B/HRV (0.24 to 0.38), HPIV/HBoV (0.21 to 0.33), HAdV/HBoV (0.26 to 0.45), and HMPV/HCoV (0.17 to 0.20). Results at *p* ≤ 0.05 and *p* ≤ 0.1 that are unadjusted for multiple comparisons are shown in Figures S9 and S10, respectively, where HPIV/HCoV showed consistent positive correlations in all four cities at *p* ≤ 0.05. Stratifying by age group, viral correlations for children were largely consistent with the overall population (Figure S11), whereas not a single virus pair was significant in a consistent direction across three cities for adults (Figure S12).

### 3.4 Seasonal and long‐term trends

To identify seasonal patterns and long‐term trends, we calculated the expected prevalence of each virus (Figure S13, Table S2). Averaging across the 11 years, the expected and observed prevalence of HMPV, IBV, RSV‐A, and RSV‐B was higher in winter months, whereas HPIV was more prevalent in spring and summer months. HAdV, HBoV, and HCoV were more variable throughout the years. Long‐term decrease in prevalence was seen for HRV in Beijing, for IAV in all cities except for Shanghai, and for IBV in Guangzhou. Long‐term increase was noted for HAdV and RSV‐A in Guangzhou and for HCoV in Shanghai.

### 3.5 Multivariate Bayesian hierarchical model

**FIGURE 4:**
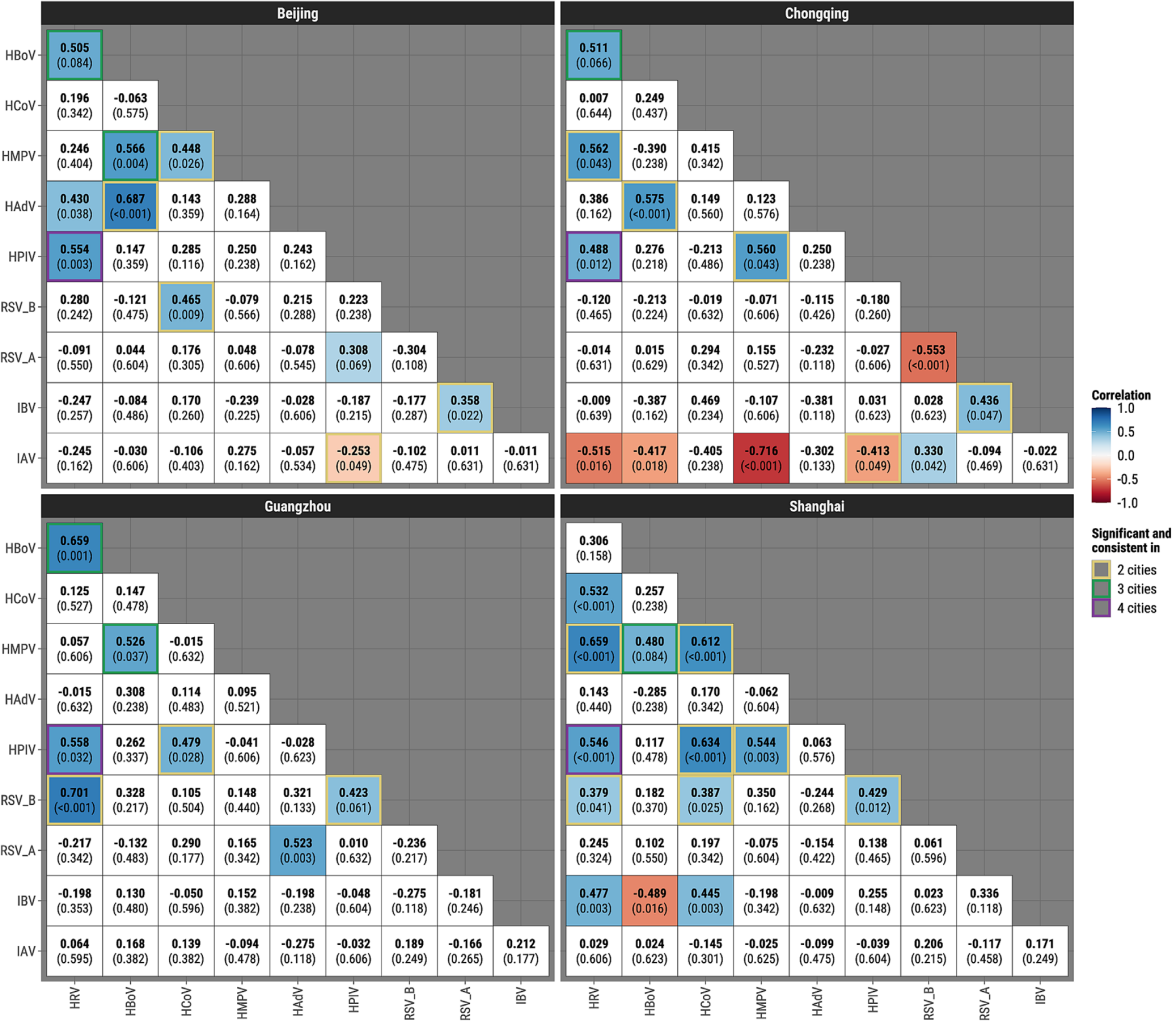
Bayesian hierarchical model correlation coefficients for the total population studied and *q‐*values, adjusting for age, sex, seasonality, changes in testing frequency, and autocorrelation. The *q*‐values represent statistical evidence adjusted for multiple comparisons by controlling the false discovery rate. Significant correlations (*q* ≤ 0.10) are shown in color. Blue and red indicate positive and negative coefficients, respectively. Yellow, green, and purple borders indicate virus pairs statistically significant with consistent directions in two, three and four cities, respectively.

After removing seasonal and long‐term trends and adjusting for multiple comparisons using *q*‐values, Bayesian hierarchical models revealed 13 significant virus–virus correlations in Shanghai (12 positive, one negative), 12 in Chongqing (seven positive, five negative), 10 (nine positive, one negative) in Beijing, and seven in Guangzhou (all positive). HPIV was positively correlated with HRV in all four cities with correlations ranging from 0.49 to 0.56 (*q*‐values varying from <0.001 to 0.032) (Figure 4, Table S3). Monthly prevalences of the two viruses across the four cities (Figure S14) confirmed this finding. There were two pairs with significant positive correlations in three cities: HBoV/HRV (0.51 to 0.66) and HBoV/HMPV (0.48 to 0.57). There was one significant negative correlation in two cities between IAV and HPIV (−0.41 to −0.25) and nine significant positive correlations in two cities: IBV/RSV‐A (0.36 to 0.44), RSV‐B/HPIV (0.42 to 0.43), RSV‐B/HCoV (0.39 to 0.47), RSV‐B/HRV (0.38 to 0.70), HPIV/HMPV (0.54 to 0.56), HPIV/HCoV (0.48 to 0.63), HAdV/HBoV (0.58 to 0.69), HMPV/HCoV (0.45 to 0.61), and HMPV/HRV (0.56 to 0.66). The highest positive correlations in each city were found between HAdV and HBoV in Beijing (*ρ* = 0.69, 95% credible interval [CI]: 0.48–0.84) and Chongqing (*ρ* = 0.58, 95% CI [0.32, 0.78]), RSV‐B and HRV in Guangzhou (*ρ* = 0.70, 95% CI [0.38, 0.90]), and HMPV and HRV in Shanghai (*ρ* = 0.66, 95% CI [0.29, 0.89]). Some viruses were interactive with as many as five other viruses in a particular city, for example, IAV in Chongqing and HRV in Shanghai. Using *p* value ≤0.05 to determine statistical significance but without adjustment for multiple comparisons (Figure S15) yielded very similar results to those based on the *q*‐values. Unadjusted analyses at the *p*‐value ≤0.1 level (Figure S16) additionally identified positive correlations between HBoV and HRV in all four cities (0.31 to 0.66).

### 3.6 Age‐stratified analysis

The Bayesian model fitted solely on children revealed many more significant virus–virus correlations than the total population, 24–29 in each of the four cities (Figure S17). All virus pairs that were significantly correlated in the total population remained significant in children, except for one pair (HBoV/HMPV) in Guangzhou. HPIV/HRV continues to be the only pair with significant correlations in all four cities (0.47 to 0.61). Furthermore, 10 virus pairs (IBV/RSV‐A [0.41 to 0.49], RSV‐A/HCoV [0.31 to 0.44], RSV‐B/HPIV [0.34 to 0.46], RSV‐B/HMPV [0.31 to 0.36], RSV‐B/HRV [0.46 to 0.67], HPIV/HMPV [0.31 to 0.62], HPIV/HCoV [0.57 to 0.60], HPIV/HBoV [0.30 to 0.33], HAdV/HBoV [0.39 to 0.73], and HBoV/HRV [0.42 to 0.61]) were positively associated and one pair (IAV/HAdV [−0.44 to −0.34]) was negatively associated in three cities. Conversely, when restricting to adults, there were no virus pairs that were significant in more than one city (Figure S18). Five, two and four virus pairs were found positively associated at the level of *q* ≤ 0.10 among adults in Beijing, Guangzhou, and Shanghai, respectively. Chongqing was not examined due to sparsity in the number of tested adults.

## 4 DISCUSSION

We assessed potential positive and negative interactions between acute respiratory viral infections among four major cities in China over an 11‐year period, adjusting for demographics, temporal autocorrelations, seasonality, long‐term trends, and multiple comparisons. The data are unique because of not only the coverage of a long time‐span and large metro‐populations but also the simultaneous testing of a comprehensive panel of respiratory viruses from each patient. The most convincing interactions, as shown by both the overall analyses and children‐specific analyses, are the positive correlations between HPIV and HRV and between HBoV and HRV. Although the pair of HBoV and HRV did not reach statistical significance in Shanghai, the direction of association is consistent in all four cities. Equally noticeable is the pair of RSV‐A and HCoV among children, which showed consistent positive associations in all four cities and statistical significance in three. More positive correlations were found than negative correlations, and more associations were found in children than in the general population. There was also a substantial level of spatial heterogeneity in the virus–virus interactions, for example, strong negative correlation of IAV with HRV and HMPV, as well as that between RSV‐A and RSV‐B, were only seen in Chongqing.

HPIV was positively correlated with HRV in all cities in Bayesian analyses. HPIVs are one of the most common causes of lower respiratory tract infections among children <5 years, accounting for up to 17% of hospitalizations from pneumonia.^17^ HRVs are the most frequent pathogens associated with viral respiratory infections among all age groups, with infants, young children, older adults, and immunocompromised at higher risk of severe disease.^18^ Although there are limited long‐term population‐level data on HPIV/HRV interactions, studies have shown HPIV and HRV often present clinically as co‐infections,^19^ consistent with our data in Beijing, Chongqing, and Guangzhou (Figure S8). HPIV, which had the most co‐infections in this study, is a slow replicating virus with long infecting times and a slow decay rate.^5^ This unique viral behavior may indeed be a contributing factor to its higher prevalence in co‐infections, as an extended presence within the host provides more opportunities for encounters with other respiratory viruses.^5^, ^19^, ^20^ This protracted viral shedding might create a milieu where HPIV and other respiratory viruses can coexist, leading to a higher likelihood of co‐infections observed in our study. Although we were unable to disaggregate HPIV by serotypes due to statistical power constraints, HPIV‐3 was predominant in this study. Viral load for HPIV‐3 was demonstrated to be consistently high regardless of co‐infection status.^21^ HRV RNA may be detected up to 5–6 weeks after onset of symptoms and 2 weeks after symptoms have disappeared.^22^ Infection with HRV, a fast growing virus,^5^ may predispose the immune system to subsequent bacterial and/or viral infections via interference with the Type‐I interferons pathway, modulation of leukocyte interactions, and changes in cytokine production.^23^

HCoV was positively correlated with HPIV in three cities for children in adjusted analyses. We are unaware of other population‐level analyses supporting these findings, but several studies reported increased frequency of co‐infection between HPIV and HCoV‐229E in Shanghai,^24^ HCoV‐NL63 in Beijing,^25^ and HCoV‐OC43 in Guangzhou.^26^ Further epidemiological studies are needed to elucidate whether the observed associations of HCoV with other respiratory viruses will bear any similarity to those of SARS‐CoV‐2 in the long run. For SARS‐CoV‐2, the effects of mass vaccinations may alter the pathological sequelae and hence its diagnosis rate and surveillance.

In adjusted analyses, we observed positive correlations among pairs of viruses including RSV‐B, HBoV, HAdV, and HMPV—viruses that frequently cause lower respiratory tract infections in young children. This is consistent with a 10‐year study in central Europe, which found that HRV/HBoV, HRV/HAdV, RSV/HBoV, RSV/HAdV, RSV/HRV, and RSV/HCoV‐OC43 were the most prevalent co‐infections.^27^ A study in central Europe that examined prevalence of 17 respiratory viruses over a 10‐year period also found that HRV/HBoV, HRV/HAdV, RSV/HBoV, RSV/HAdV, RSV/HRV, and RSV/HCoV‐OC43 were the most prevalent co‐infections.^27^ Children, particularly those <5 years, are known to be more susceptible to viral co‐infections than adults possibly due to higher contact frequencies with peers and/or immune system immaturity.^28^

Among children, IAV was negatively correlated with HAdV in three cities, and HRV, HBoV, HMPV, and RSV‐B in Chongqing, which is in accord with several studies that reported shifts in the timing and severity of other respiratory infections following IAV epidemics.^11^, ^12^ These negative correlations are unlikely to be related to immune responses induced by influenza vaccines as recent studies suggest seasonal influenza vaccination may not provide the same level of non‐specific immunity,^29^ and influenza vaccination coverage in China is generally low.^30^ The unique geographic and environmental characteristics of Chongqing could have played a role in the more prominent negative virus–virus associations involving IAV. Chongqing has a subtropical monsoon climate and very high relative humidity. Our study showed higher test‐positive rates for RSV‐A, RSV‐B, and HRV among children in Chongqing compared with the other three cities, consistent with results from a study at a children's hospital in Chongqing during 2013–2018.^31^ Meteorological factors in Chongqing have been shown to be associated with influenza and pertussis.^32^, ^33^

Most of the statistically significant correlations among respiratory viruses were positive associations, which could result from several factors, including shared transmission routes, interactions with the host immune system, or environmental conditions that favor multiple virus types.^34^, ^35^ It is also conceivable that variations across years in the drivers of transmission common to several viruses, such as unusual climate conditions, social contact patterns, or changes in healthcare‐seeking behavior, could cause multiple viruses' incidences to deviate similarly from their expected seasonal periodicity. This synchronized deviation might have contributed to the observed positive correlations among virus pairs.

There were inconsistencies in virus–virus interactions between adult and child populations, even within the same city. Children and adults have distinct immune responses due to differences in immune system maturity and prior exposure to respiratory viruses.^36^ Children often have less developed immune systems and may not have encountered certain viruses previously, rendering them more susceptible to infection and disease. Conversely, adults may have acquired partial immunity over time, leading to variations in co‐infection rates. The contact patterns and behaviors of children and adults also differ significantly. Children typically engage in close contact in school and daycare settings, whereas adults may have workplace interactions and different social behaviors. Additionally, healthcare‐seeking behaviors and testing practices can vary between age groups. Parents may be more likely to seek medical care for young children, resulting in increased testing rates for pediatric populations.

Our study had several limitations. First, we did not assess higher order interactions between three or more viruses. Second, bacterial infections, which were not examined in this study, may also play a mediating role in one's susceptibility to viral co‐infection. Third, 4.9% of patients included in this study were only tested for two viruses of interest (IAV and IBV) during the 2009 H1N1 influenza pandemic, suggesting some co‐infections in those patients might have been missed. Fourth, our study used hospital surveillance data, which includes symptomatic individuals seeking care. Influenza, RSV, and other viral infections may not present clinically, so it is unclear to what extent asymptomatic infections impacted the observed interactions.^37^ Additionally, co‐infections may be more clinically severe and hence more likely to be identified than single infections. Fifth, heterogeneity in test‐positive rates and virus–virus correlations across cities may be attributed in part to differential healthcare‐seeking behavior, testing practices and other socioeconomic factors rather than biological interactions between viruses. In addition, the Bayesian modeling did not consider sensitivity and specificity of the testing methods. Finally, while detrending seasonality and long‐term patterns in the step of calculating expected infection frequencies is to minimize the impact of population‐level confounders such as human mobility and vaccination policies, some biological mechanisms driving virus–virus interactions may also be modulated by seasonality, and therefore, detrending could have masked some of these mechanisms.

## 5 SUMMARY

Virus–virus interactions may have a significant impact on viral disease severity, transmissibility, immune response, and vaccine effectiveness. Different virus pairings may trigger different pathogenic mechanisms, which could inhibit or amplify clinical and public health consequences. Spatially consistent virus–virus interactions warrant future research to understand their biological pathways and social determinants and may be considered for more effective intervention programs, for example, developing combination vaccines for children. Our method extends the statistical approach designed for dense sampling to sparse sampling to provide insights of virus–virus interactions among four major cities in China over an 11‐year period. The method could be further improved by incorporating individual‐level co‐infection status. The SARS‐CoV‐2 pandemic may pose new challenges on modeling virus–virus interactions. Recent research demonstrates a significant reduction in a broad spectrum of acute respiratory viruses consequent to SARS‐CoV‐2 likely due to the synergy of nonpharmaceutical interventions (hand hygiene, social distancing, and facemask use), reduced testing of seasonal respiratory viruses, and viral interferences.^38^ Testing patients for multiple respiratory pathogens whenever resources permit will improve our understanding of interactions among the pathogens, clinical treatment of co‐infections, and evaluation of intervention and prevention strategies.

## AUTHOR CONTRIBUTIONS

**Zachary J. Madewell:** Formal analysis; investigation; methodology; software; validation; visualization; writing—original draft; writing—review and editing. **Li‐ping Wang:** Conceptualization; data curation; funding acquisition; investigation; methodology; project administration; resources; writing—review and editing. **Natalie E. Dean:** Funding acquisition; investigation; methodology; writing—original draft; writing—review and editing. **Hai‐Yang Zhang:** Data curation; investigation; methodology; resources; writing—review and editing. **Yi‐Fei Wang:** Data curation; investigation; methodology; resources; writing—review and editing. **Xiao‐Ai Zhang:** Data curation; investigation; methodology; resources; writing—review and editing. **Wei Liu:** Data curation; investigation; methodology; resources; writing—review and editing. **Wei‐Zhong Yang:** Data curation; funding acquisition; investigation; methodology; resources; writing—review and editing. **Ira M. Longini:** Funding acquisition; investigation; methodology; writing—review and editing. **George F. Gao:** Data curation; funding acquisition; investigation; methodology; resources; writing—review and editing. **Zhong‐Jie Li:** Data curation; funding acquisition; investigation; methodology; resources; writing—review and editing. **Li‐Qun Fang:** Conceptualization; data curation; funding acquisition; investigation; methodology; project administration; resources; supervision; validation; visualization; writing—review and editing. **Yang Yang:** Conceptualization; formal analysis; funding acquisition; investigation; methodology; project administration; resources; software; supervision; validation; visualization; writing—original draft; writing—review and editing.

## CONFLICT OF INTEREST STATEMENT

The authors declare no conflict of interests.

## ETHICS STATEMENT

Data collection was considered to be public health surveillance by the National Health Commission of the People's Republic of China and verbal informed consent was obtained from patients or their legal guardians. The surveillance protocol was reviewed and approved by the ethics review committees of the China CDC (2015‐025).

### PEER REVIEW

The peer review history for this article is available at https://www.webofscience.com/api/gateway/wos/peer-review/10.1111/irv.13212.

## Supporting information


**Figure S1.** Example MCMC trace plots of model parameter estimates for the Bayesian hierarchical model for influenza B virus (IBV) and human parainfluenza virus (HPIV) in Beijing.
**Figure S2.** Monthly numbers of laboratory‐tested samples with and without viral infections from January 2009 to December 2019 in four metro cities, China.
**Figure S3.** Monthly percentage of patients diagnosed with a single viral infection, a viral co‐infection, or determined to be virus‐negative from January 2009 to December 2019 in four metro cities, China.
**Figure S4.** Monthly viral prevalence by sex from January 2009 to December 2019 in four metro cities, China. Weighted Pearson's coefficients in monthly infection prevalence between the two sexes for each virus in each city and 95% confidence intervals are shown, with weights corresponding to the number of tests administered by city. Prevalence was the number of infected patients over the total number of patients tested for each virus for each month.
**Figure S5.** The proportion of all tests for each virus that were positive and total number of infections in four metro cities, China. Larger circles represent a greater proportion of positive tests and darker colors represent greater numbers of infections.
**Figure S6.** Number of tests (light) and proportion of tests that were positive (dark) for each acute respiratory virus by age group in four metro cities, China.
**Figure S7.** Relative prevalence of viruses from January 2009 to December 2019 in four metro cities, China.
**Figure S8.** Ratio of odds of infection with one virus between presence vs. absence of the other virus for each virus pair, based on tabulating individual detection data from January 2009 to December 2019 in four metro cities, China. Blue represents odds ratios (OR) > 1, red represents ORs < 1, and larger circles represent smaller *q*‐values. The *q*‐values control for false discovery rate and are based on *p*‐values from the Fisher's exact test.
**Figure S9.** Weighted Pearson's correlation coefficients for the total population studied and 95% confidence intervals of monthly prevalence for each pair of respiratory viruses in four metro cities of China, where weights are the numbers of tests administered. This figure is identical to Figure 3, but with 95% confidence intervals instead of *q‐*values. Blue and red indicate statistically significant (*p* < 0.05) positive and negative coefficients, respectively. Green and purple borders indicate virus pairs significant in three and four cities, respectively. The *p*‐values are not adjusted for multiple comparisons.
**Figure S10.** Weighted Pearson's correlation coefficients for the total population studied and 90% confidence intervals of monthly prevalence for each pair of respiratory viruses in four metro cities of China, where weights are the numbers of tests administered. This figure is identical to Figure 3, but with 90% confidence intervals instead of *q‐*values. Blue and red indicate statistically significant (*p* < 0.1) positive and negative coefficients, respectively. Green and purple borders indicate virus pairs significant in three and four cities, respectively. The *p*‐values are not adjusted for multiple comparisons.
**Figure S11.** Weighted Pearson's correlation coefficients for children <18 years and *q*‐values of monthly prevalence for each pair of respiratory viruses in four metro cities of China, where weights are the numbers of tests administered. The *q*‐values represent statistical evidence adjusted for multiple comparisons by controlling the false discovery rate. Significant correlations (*q* ≤ 0.10) are shown in colour. Blue and red indicate positive and negative coefficients, respectively. Green and purple borders indicate virus pairs statistically significant with consistent directions in three and four cities, respectively.
**Figure S12.** Weighted Pearson's correlation coefficients for adults ≥18 years and *q*‐values of monthly prevalence for each pair of respiratory viruses in four metro cities of China, where weights are the numbers of tests administered. The *q*‐values represent statistical evidence adjusted for multiple comparisons by controlling the false discovery rate. Significant correlations (*q* ≤ 0.10) are shown in colour. Blue and red indicate positive and negative coefficients, respectively. Yellow borders indicate virus pairs statistically significant with consistent directions in two of the three cities, respectively. There was a lower testing rate for adults in Chongqing, therefore that subgroup was not analyzed.
**Figure S13.** The observed and expected prevalence of each virus before applying the multivariate Bayesian hierarchical model, January 2009 to December 2019 in four metro cities, China. The expected prevalence was calculated by fitting generalized linear models for each virus and city with harmonic functions to account for seasonality and polynomials to account for long‐term trends, while adjusting for sex and age group (categorized as <5, ≥5–18, ≥18–40, and ≥40 years). Prevalence was the number of infected patients over the total number of patients tested for each virus for each month.
**Figure S14.** Monthly prevalence of human parainfluenza virus and human rhinovirus from January 2009 to December 2019 in four metro cities, China. Prevalence was the number of infected patients over the total number of patients tested for each virus for each month.
**Figure S15.** Bayesian hierarchical model correlation coefficients for the total population studied and 95% credible intervals, adjusting for age, sex, seasonality, changes in testing frequency, and autocorrelation. This figure is identical to Figure 4, but with 95% credible intervals instead of *q‐*values. Blue and red indicate statistically significant (*p* < 0.05) positive and negative coefficients, respectively. Purple borders indicate virus pairs significant in four cities. The *p*‐values are not adjusted for multiple comparisons.
**Figure S16.** Bayesian hierarchical model correlation coefficients for the total population studied and 90% credible intervals, adjusting for age, sex, seasonality, changes in testing frequency, and autocorrelation. This figure is identical to Figure 4, but with 90% credible intervals instead of *q‐*values. Blue and red indicate statistically significant (*p* < 0.10) positive and negative coefficients, respectively. Green and purple borders indicate virus pairs significant in three and four cities, respectively. The *p*‐values are not adjusted for multiple comparisons.
**Figure S17.** Bayesian hierarchical model correlation coefficients for children <18 years and *q*‐values, adjusting for age, sex, seasonality, changes in testing frequency, and autocorrelation. The *q*‐values represent corrected *p*‐values for multiple comparisons by controlling the false discovery rate. Significant correlations (*q* ≤ 0.10) are shown in color. Blue and red indicate positive and negative coefficients, respectively. Green and purple borders indicate virus pairs significant in three and four cities, respectively.
**Figure S18.** Bayesian hierarchical model correlation coefficients for adults ≥18 years and *q*‐values, adjusting for age, sex, seasonality, changes in testing frequency, and autocorrelation. The *q*‐values represent statistical evidence adjusted for multiple comparisons by controlling the false discovery rate. Significant correlations (*q* ≤ 0.10) are shown in colour. Blue and red indicate positive and negative coefficients, respectively. There was a lower testing rate for adults in Chongqing, therefore that subgroup was excluded from this analysis.
**Table S1.** Numbers of viruses tested for each patient (N = 101 643). Viruses tested included: influenza A and B; respiratory syncytial virus A and B; and human parainfluenza virus, adenovirus, metapneumovirus, coronavirus, bocavirus, and rhinovirus.
**Table S2.** Quasi‐Akaike information criteria for harmonic regression models for the total population studied by virus and city. The lowest Quasi‐Akaike information criteria for each model is shown in bold.
**Table S3.** Mean, standard deviation, and quantiles of the marginal posterior distribution for *ρ*, and convergence diagnostics for Bayesian hierarchical model.

## Data Availability

The datasets used and/or analyzed during the current study are available from the corresponding author on reasonable request.
