# Supplementary material for: Interactions among acute respiratory viruses in Beijing, Chongqing, Guangzhou, and Shanghai, China, 2009–2019
Source: Influenza Other Respir Viruses. 2023 Nov 12;17(11):e13212. doi: 10.1111/irv.13212 (PMC10640964; doi:10.1111/irv.13212)
Supplement: Supplementary file 1 — Figure S1. Example MCMC trace plots of model parameter estimates for the Bayesian hierarchical model for influenza B virus (IBV) and human parainfluenza virus (HPIV) in Beijing. Figure S2. Monthly numbers of laboratory‐tested samples with and without viral infections from January 2009 to December 2019 in four metro cities, China. Figure S3. Monthly percentage of patients diagnosed with a single viral infection, a viral co‐infection, or determined to be virus‐negative from January 2009 to December 2019 in four metro cities, China. Figure S4. Monthly viral prevalence by sex from January 2009 to December 2019 in four metro cities, China. Weighted Pearson's coefficients in monthly infection prevalence between the two sexes for each virus in each city and 95% confidence intervals are shown, with weights corresponding to the number of tests administered by city. Prevalence was the number of infected patients over the total number of patients tested for each virus for each month. Figure S5. The proportion of all tests for each virus that were positive and total number of infections in four metro cities, China. Larger circles represent a greater proportion of positive tests and darker colors represent greater numbers of infections. Figure S6. Number of tests (light) and proportion of tests that were positive (dark) for each acute respiratory virus by age group in four metro cities, China. Figure S7. Relative prevalence of viruses from January 2009 to December 2019 in four metro cities, China. Figure S8. Ratio of odds of infection with one virus between presence vs. absence of the other virus for each virus pair, based on tabulating individual detection data from January 2009 to December 2019 in four metro cities, China. Blue represents odds ratios (OR) > 1, red represents ORs < 1, and larger circles represent smaller q‐values. The q‐values control for false discovery rate and are based on p‐values from the Fisher's exact test. Figure S9. Weighted Pearson's correlation coeff [file IRV-17-e13212-s001.docx]

**Supplementary Materials**

**Interactions among acute respiratory viruses in Beijing, Chongqing, Guangzhou, and Shanghai, China, 2009-2019**

**Supplementary Methods**

*Multivariate Bayesian Hierarchical Model*

Simple correlations may be confounded by changes in testing frequencies, temporal autocorrelation, and other factors, so we adapted a multivariate Bayesian hierarchical model designed by Mair *et al.* [1] to adjust for potential confounding. As second moments such as correlations are much more difficult to estimate than first moments, a two-stage approach is used. Before applying the hierarchical model, we first smoothed observed monthly infection counts for each virus using logistic regression, adjusting for common risk factors for infection. Mair *et al*. fitted mixed effect logistic models to the individual data for each virus and calendar month separately, with age and sex as fixed effects and year as a random effect. This method, while adequately accounting for variation from month to month, may absorb a lot of variations that may inform virus-virus interactions into the expected values (first moment). Instead of using month-specific models, we fit an overall generalized linear model for each virus and city with harmonic functions to account for seasonality and polynomials of year to account for long-term trends, while adjusting for sex and age group (categorized as <5, ≥5-18, ≥18-40, and ≥40 years). Our models are more restrictive in the mean structure to allow more signals to be explained by the random effects in the Bayesian hierarchical model for the correlations. To account for the excessive number of zeros, we used a quasi-binomial model of the general form:

$$\begin{matrix} Z_{ymiv}\sim\text{quasi-Binomial}( \pi_{ymiv}) \\ log\left( \frac{\pi_{ymiv}}{1-\pi_{ymiv}} \right)=\beta_{v0}+\beta_{v1}{SEX}_{ymi}+{\boldsymbol{\beta}_{v2}}^{'}\boldsymbol{AGE}_{\boldsymbol{ymi}}+{\boldsymbol{\beta}_{v3}}^{'}\boldsymbol{SEASON}_{m}+{\boldsymbol{\beta}_{v4}}^{'}\boldsymbol{POLY}_{y} \end{matrix}$$

where $Z_{ymiv}$ is the test outcome (1=positive, 0=negative) with regard to virus $v$ for the $i^{th}$ sample during month $m$ of year $y$. ${SEX}_{ymi}$ indicates the sex and $\boldsymbol{AGE}_{\boldsymbol{ymi}}$ is a vector of indicators for age groups, seasonal oscillations in the outcomes are described by harmonic functions $\boldsymbol{SEASON}_{m}=\left( sin\left( \frac{2\pi m}{12} \right), cos\left( \frac{2\pi m}{12} \right), sin\left( \frac{2^{2}\pi m}{12} \right),cos\left( \frac{2^{2}\pi m}{12} \right) \right)^{'}$, and long term yearly trend is captured by the polynomial vector $\boldsymbol{POLY}_{y}=\left( y, y^{2} \right)^{'}$. $\beta_{v0}$, $\beta_{v1}$, $\boldsymbol{\beta}_{v2}$, $\boldsymbol{\beta}_{v3}$ and $\boldsymbol{\beta}_{v4}$ are the corresponding coefficients. We evaluated six models with harmonic functions (up to four cycles per year) and polynomials (up to quadratic) of year to best capture yearly and seasonal variations and selected the model with the lowest quasi-Akaike information criteria (Table S2). The expected prevalence of positive samples associated with virus $v$, month $m$ and year $y$ is simply $\hat{\pi}_{ymv}=\frac{1}{N_{ymv}}\sum_{i=1}^{N_{ymv}} \hat{\pi}_{ymiv}$, where $\hat{\pi}_{ymiv}$ is the model fitted probability for each sample.

In the second stage, we model each pair of viruses separately. We assume the realized prevalence of positive samples, $\pi_{ymv}^{*}$, deviated from the expected prevalence, $\hat{\pi}_{ymv}$, by a multiplicative random odds ratio (OR) for each virus,

$$\frac{\pi_{ymv}^{*}}{1-\pi_{ymv}^{*}}={OR}_{mtv}\times\frac{\hat{\pi}_{ymv}}{1-\hat{\pi}_{ymv}}$$

$${OR}_{ymv}=e^{\varphi_{ymv}-0.5\sigma_{v}^{2}}$$

The random odds ratio ${OR}_{ymv}$ is the exponentiated normally distributed random effect $\varphi_{mtv}$ with mean 0 and marginal variance $\sigma_{v}^{2}$. The subtraction of $0.5\sigma_{v}^{2}$ from $\varphi_{ymv}$ ensures ${OR}_{ymv}$ has a mean 1. $\varphi_{ymv}$ is an element in a vector of random effects modelled through a multivariate AR(1) structure:

$\varphi_{y..}|\varphi_{(y-1)..}\sim\mathrm{MVN}(s_{v}\varphi_{\left( y-1 \right)..},\left[ \Omega\bigotimes\Lambda\right]^{-1})$.

The vector of random effects $\varphi_{y..}$ is organized by nesting virus types within each month, i.e., $\varphi_{y..}=(\varphi_{y11}, \varphi_{y12},\varphi_{y21}, \varphi_{y22},\ldots,\varphi_{y\left( 12 \right)1}, \varphi_{y\left( 12 \right)2})'$ which depends on $\varphi_{(y-1)..}$ of the previous year, and $s_{v}<1$ is the autoregression coefficient. The precision matrix $\Omega=D-\lambda W$ captures the correlation of the random effects between months via the neighborhood matrix $W={[w_{ij}]}_{12\times12}$, where $w_{ij}=1$ if months $i$ and $j$ are considered neighbors and 0 otherwise. $\lambda$ is the smooth parameter which is set to 0.2 as it seems not identifiable in a preliminary simulation study. $D$ is a diagonal matrix with the diagonal element $D_{ii}=\sum_{j=1}^{12} w_{ij}$. Different from [1], $W$ is not intended to capture seasonality in virus infection frequencies because seasonality has already been accounted for in the mean trend of $\hat{\pi}_{ymv}$. We therefore used a shorter neighbourhood dependency structure, where the neighborhood for each month is composed of the flanking four months, two before and two after. Let $\Lambda={[\lambda_{ij}]}_{2\times2}$ be the precision matrix for the marginal bivariate normal distribution of $(\varphi_{ym1}, \varphi_{ym2})$. $\Lambda^{-1}$ is the between-virus covariance matrix, and the corresponding correlation matrix is of the primary inferential interest as it describes the interaction between the viruses at the population level. A usual decomposition of the covariance matrix is

$$\Lambda^{-1}=\Sigma\Gamma\Gamma^{T}\Sigma$$

where $\Sigma$ is a diagonal matrix of standard deviations and $\Gamma$ is the Cholesky decomposition of the correlation matrix and is a lower-triangular matrix. We use an alternative but equivalent parameterization by letting $\Gamma=\left( \begin{matrix} 1 & 0 \\ \rho& 1 \end{matrix} \right)$ and $\Sigma=\left( \begin{matrix} \sigma_{1} & 0 \\ 0 & \sigma_{2} \end{matrix} \right)$. This way, the marginal variances of and correlation coefficient between the two viruses are $\sigma_{1}^{2}$, $\sigma_{2}^{2}(1+\rho^{2})$ and $\rho/{\sqrt{1+\rho^{2}}}$, respectively. We report the posterior results of $\rho/{\sqrt{1+\rho^{2}}}$ to characterize the virus-virus interaction. Although the theoretical range of $\rho$ is ($-\infty,\infty$), a range of (-3, 3) for $\rho$ can effectively cover a range of (-0.95, 0.95) for the correlation coefficient. We therefore assign to $\rho$ a truncated normal prior, $N(0,1)I_{[-3,3]}$. We did not use a flat prior so that $\rho/{\sqrt{1+\rho^{2}}}$ is pulled away from 0 only when data contain a strong signal.

Given $\pi_{ymv}^{*}$ and $N_{ymv}$, we can specify the distribution of $N_{ymv}^{+}$, the number of positive tests for virus $v$ in month $m$ of year $y$. A fundamental difference in our model, as compared to the Poisson model used by Mair *et al.* [1], is that we used a zero-inflated binomial model for the monthly number of positive tests in order to account for the excess zeros and the small number of tests in some months. Finally, $N_{ymv}^{+}$ was modelled as:

$$N_{ymv}^{+}\sim Binomial(N_{ymv}, \pi_{ymv})$$

$$\pi_{myv}=\pi_{myv}^{*}U_{ymv}$$

$$U_{ymv}\sim Bernoulli(p_{v})$$

where $U_{mtv}$ is an extra Bernoulli random variable to add zero-inflation, and $p_{v}$ is the associated probability which differs by virus. When $U_{mtv}=0$, $N_{ymv}^{+}=0$; when $U_{mtv}=0$, $N_{ymv}^{+}\sim Binomial(N_{ymv}, \pi_{myv}^{*})$. Consequently, $N_{ymv}^{+}$ has an extra probability of $1-p_{v}$ to be 0.

We obtained the (2.5%, 97.5%) and (5%, 95%) sample quantiles of the posterior samples of the correlation coefficient $\rho/{\sqrt{1+\rho^{2}}}$ as its 95% and 90% credible intervals, respectively. To derive asymptotic 2-sided *p*-values, we calculated Bayesian z-scores (posterior means divided by posterior standard deviations) which were compared with the probability density function of the standard normal distribution. These *p*-values were used to derive *q*-values which account for multiple comparisons by controlling the false discovery rate (FDR). *q*-values <0.10 are considered statistical evidence.

We used ten chains, a burn-in of 20,000 iterations, and 600 thinned draws from 60,000 additional iterations across each chain with clear convergence (Figure S1).

**Reference**

1. Mair C, Nickbakhsh S, Reeve R, McMenamin J, Reynolds A, Gunson RN, et al. Estimation of temporal covariances in pathogen dynamics using Bayesian multivariate autoregressive models. *PLOS Computational Biology*. 2019;15(12):e1007492.

**
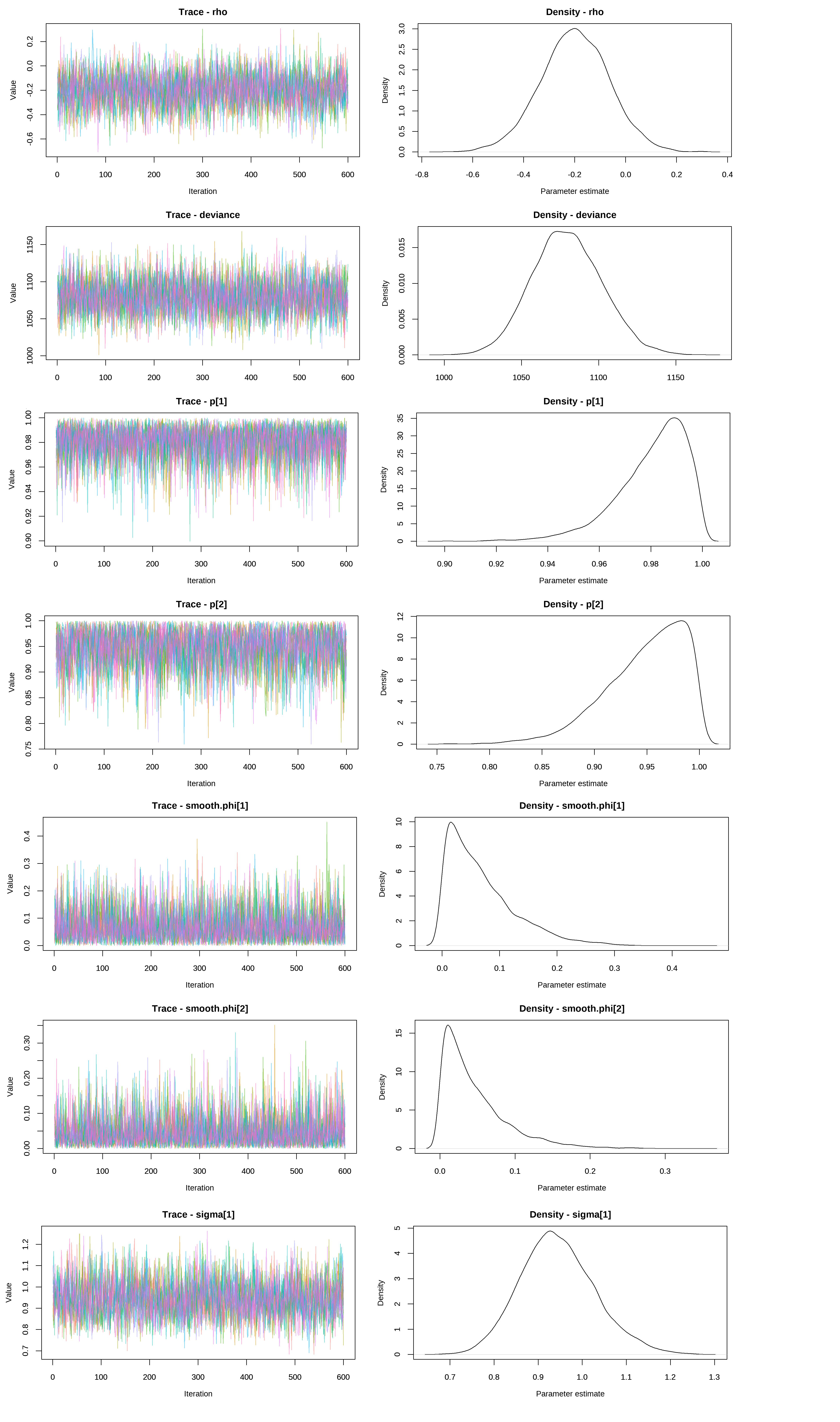
**

**Figure S1**. Example MCMC trace plots of model parameter estimates for the Bayesian hierarchical model for influenza B virus (IBV) and human parainfluenza virus (HPIV) in Beijing.


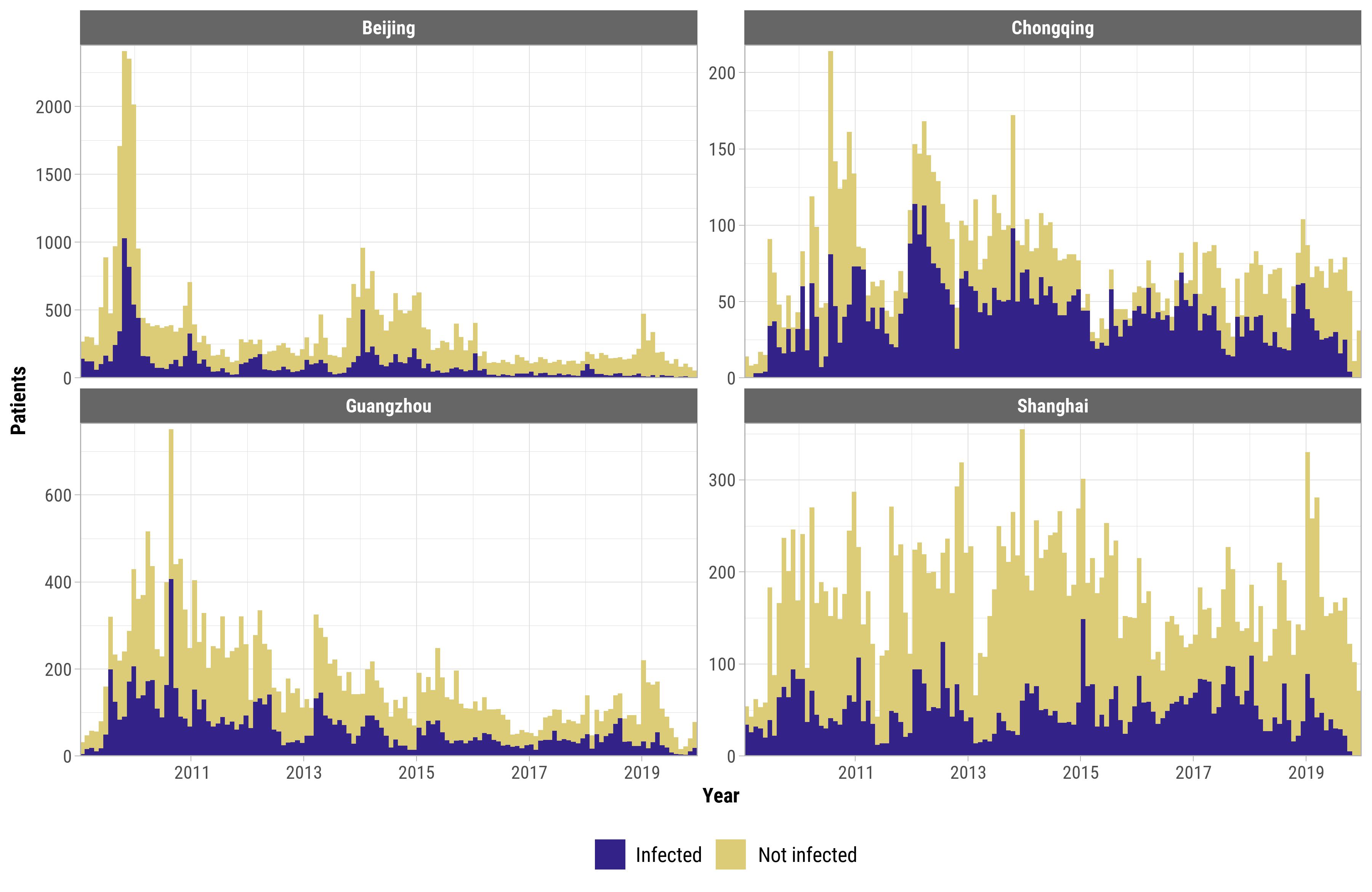


**Figure S2**. Monthly numbers of laboratory-tested samples with and without viral infections from January 2009 to December 2019 in four metro cities, China.


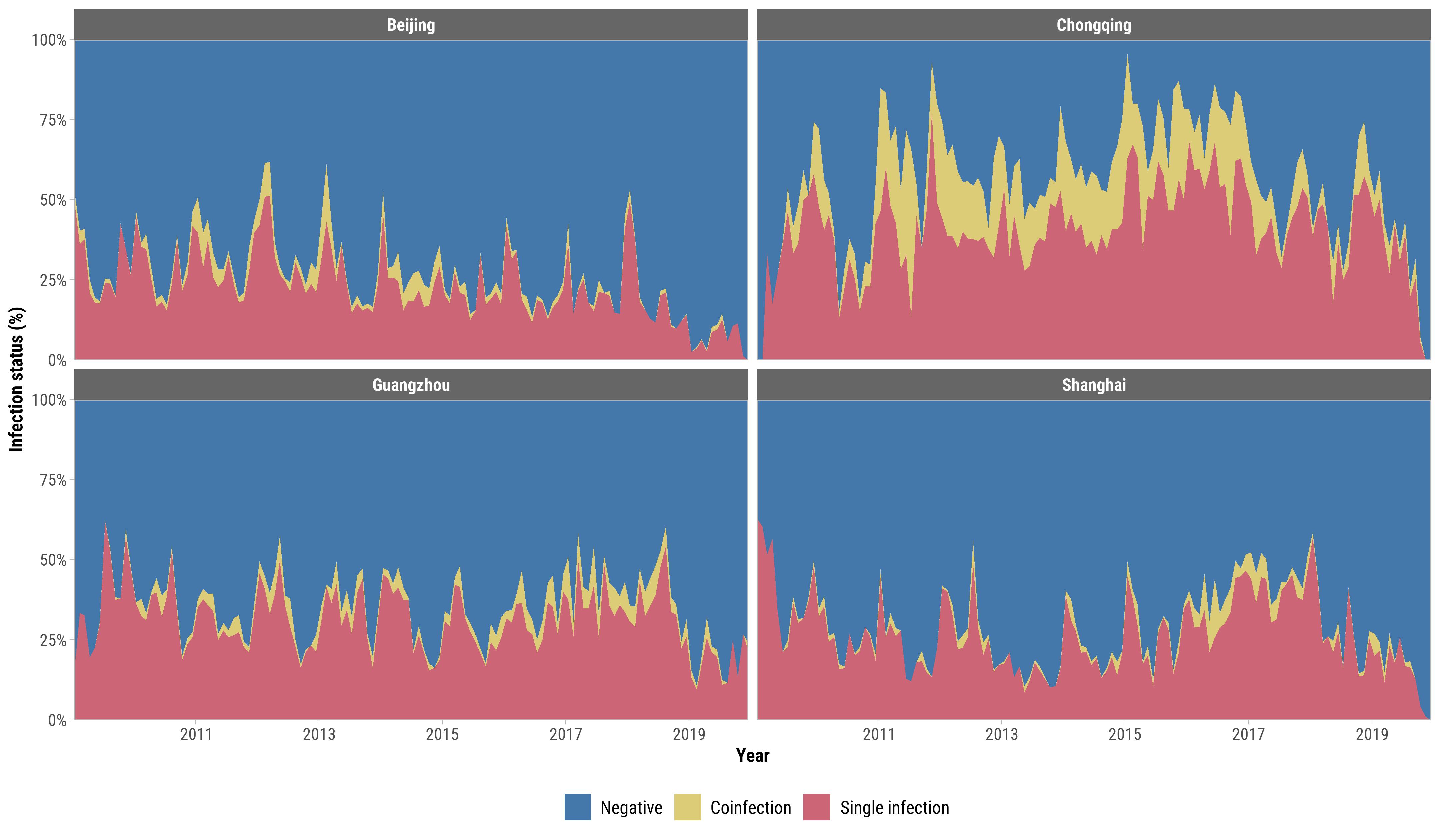


**Figure S3**. Monthly percentage of patients diagnosed with a single viral infection, a viral co-infection, or determined to be virus-negative from January 2009 to December 2019 in four metro cities, China.


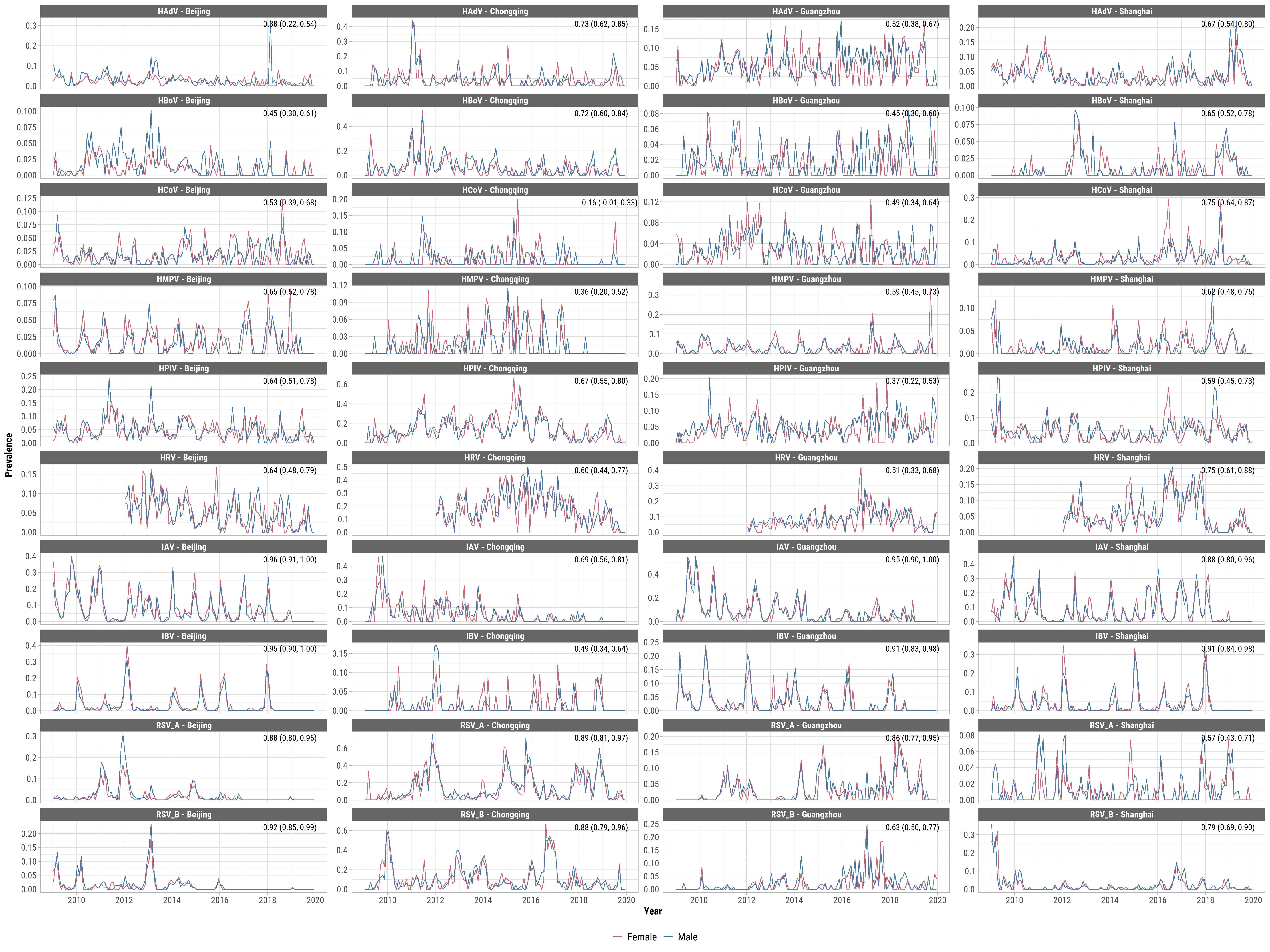
**Figure S4**. Monthly viral prevalence by sex from January 2009 to December 2019 in four metro cities, China. Weighted Pearson’s coefficients in monthly infection prevalence between the two sexes for each virus in each city and 95% confidence intervals are shown, with weights corresponding to the number of tests administered by city. Prevalence was the number of infected patients over the total number of patients tested for each virus for each month.


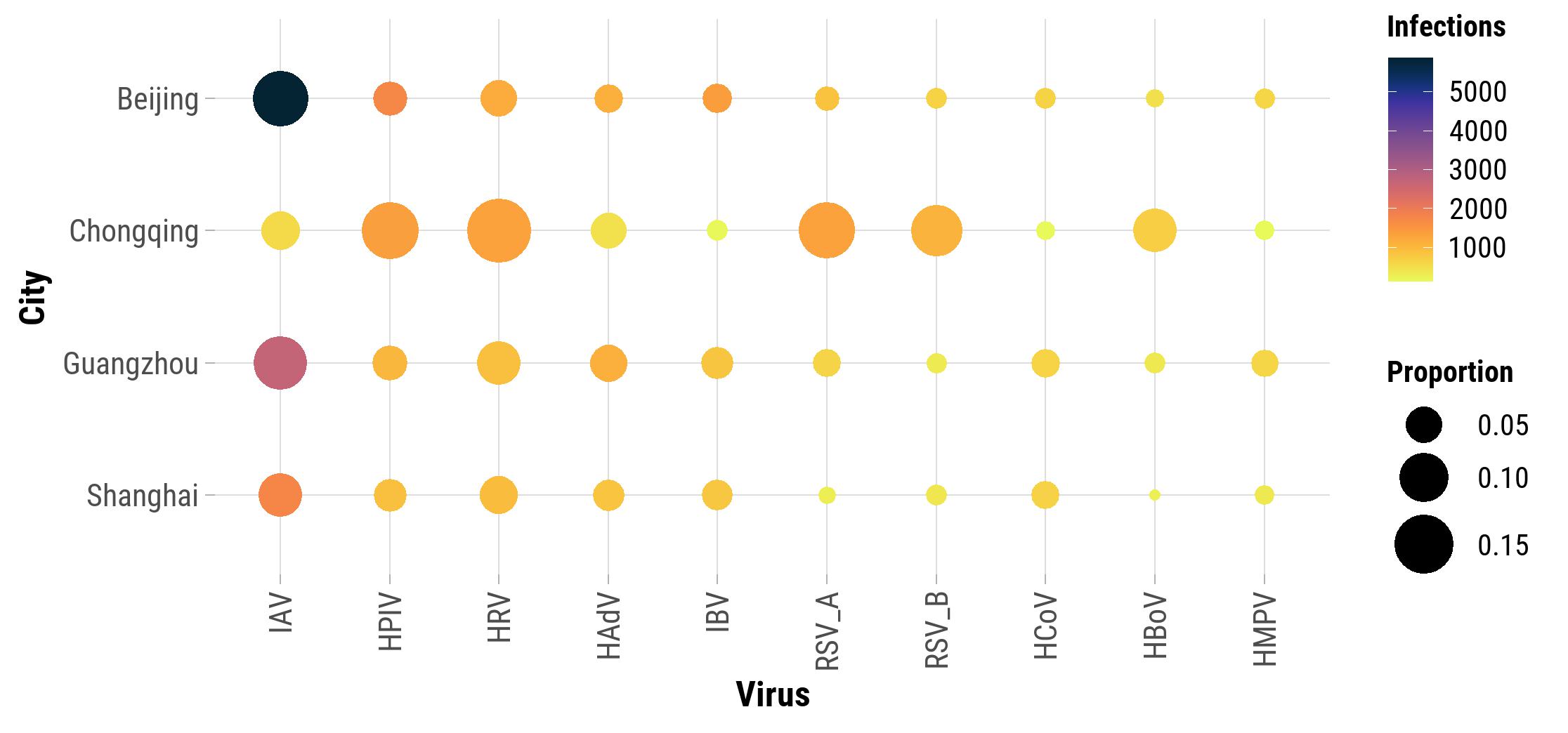


**Figure S5**. The proportion of all tests for each virus that were positive and total number of infections in four metro cities, China. Larger circles represent a greater proportion of positive tests and darker colors represent greater numbers of infections.


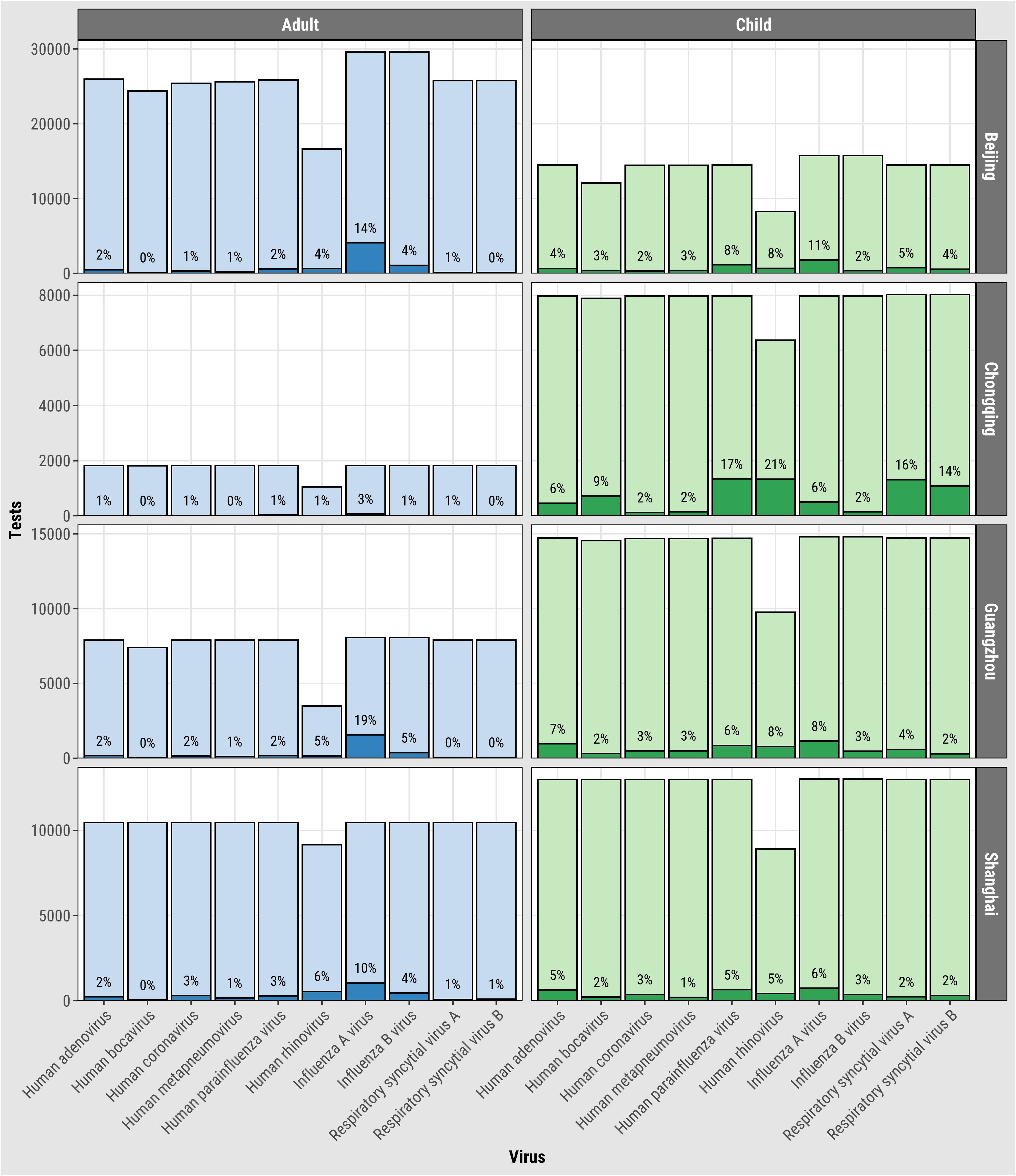


**Figure S6**. Number of tests (light) and proportion of tests that were positive (dark) for each acute respiratory virus by age group in four metro cities, China.


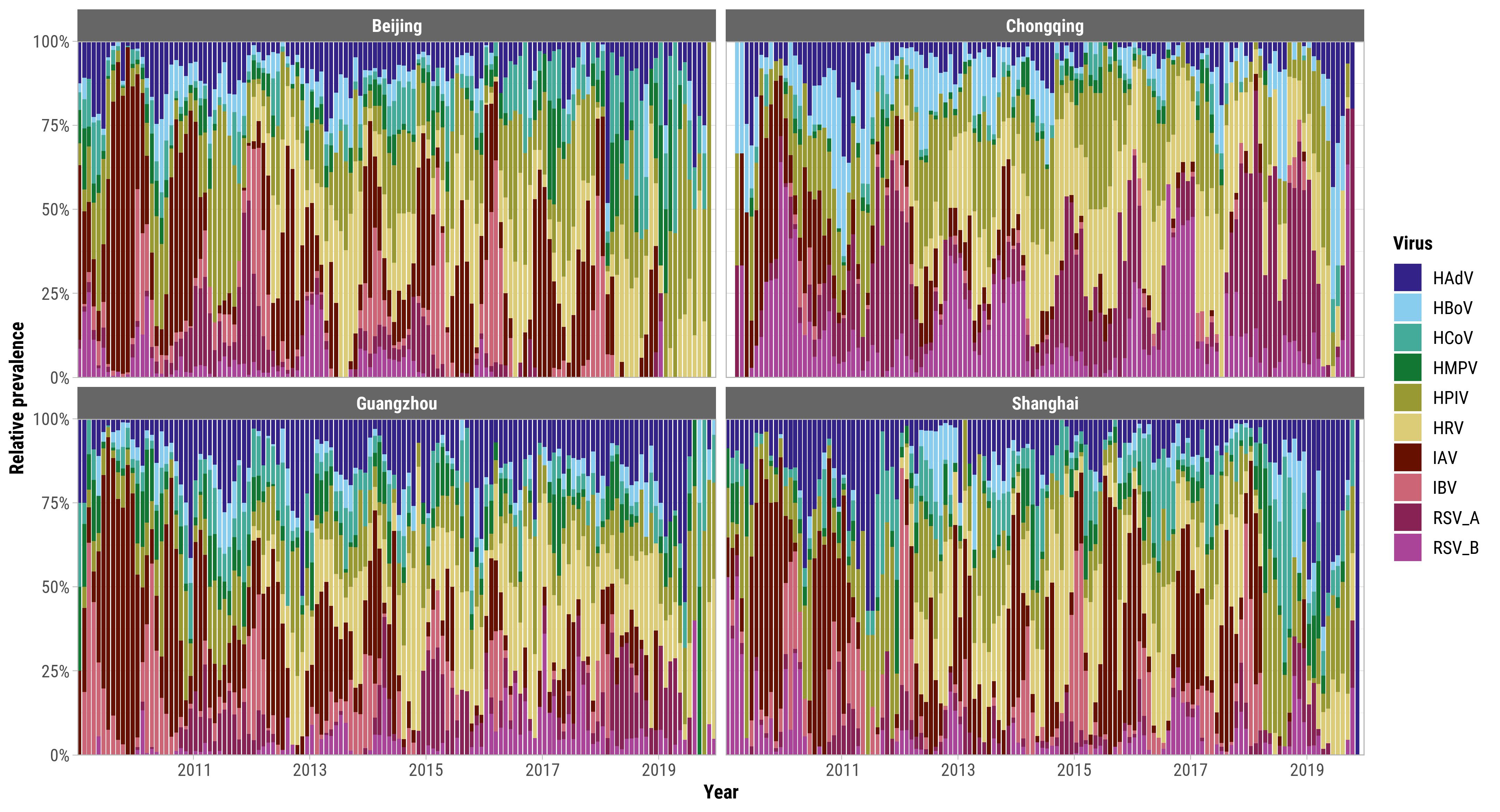


**Figure S7**. Relative prevalence of viruses from January 2009 to December 2019 in four metro cities, China.


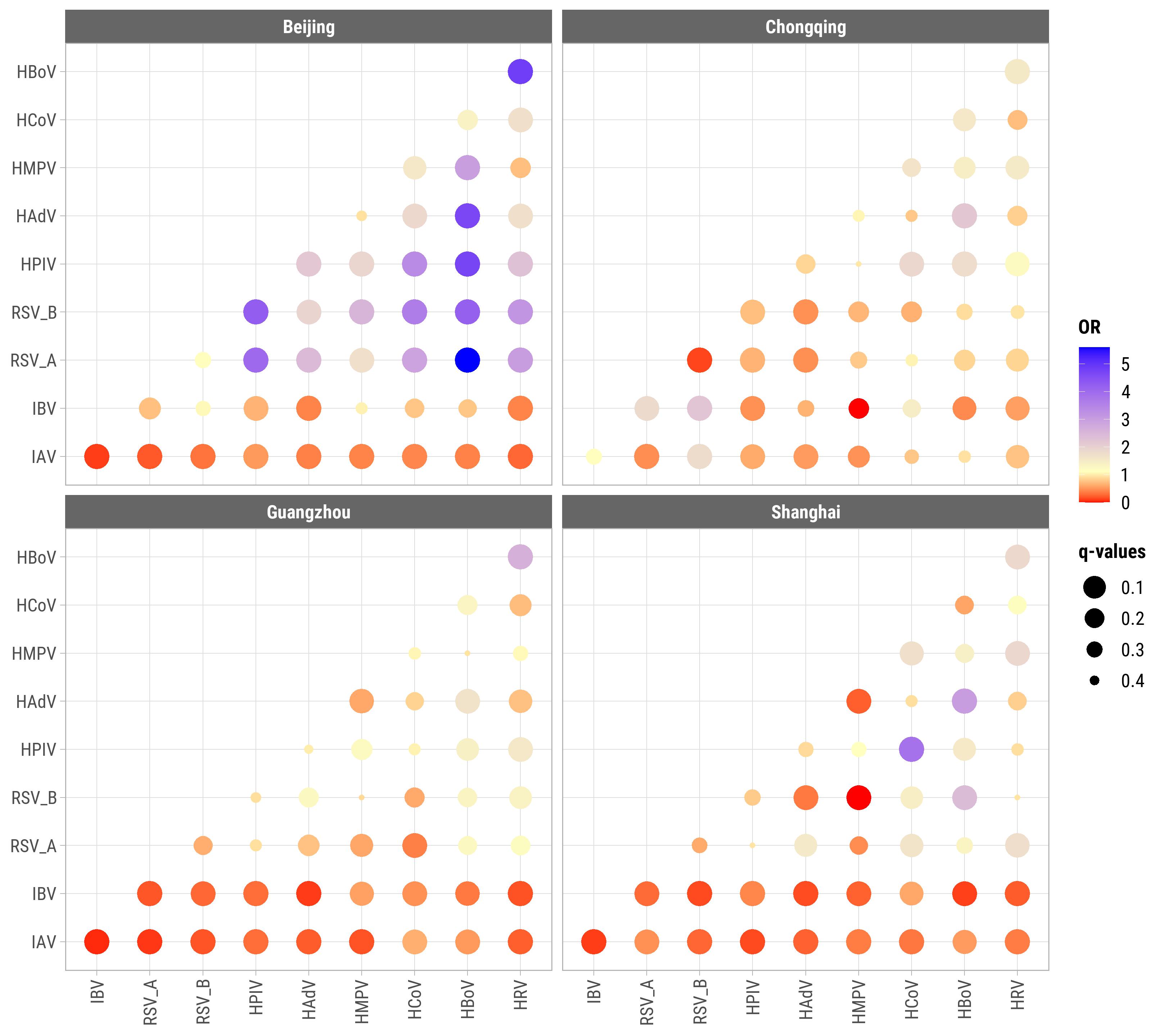


**Figure S8**. Ratio of odds of infection with one virus between presence vs. absence of the other virus for each virus pair, based on tabulating individual detection data from January 2009 to December 2019 in four metro cities, China. Blue represents odds ratios (OR) >1, red represents ORs <1, and larger circles represent smaller *q*-values. The *q*-values control for false discovery rate and are based on *p*-values from the Fisher’s exact test.


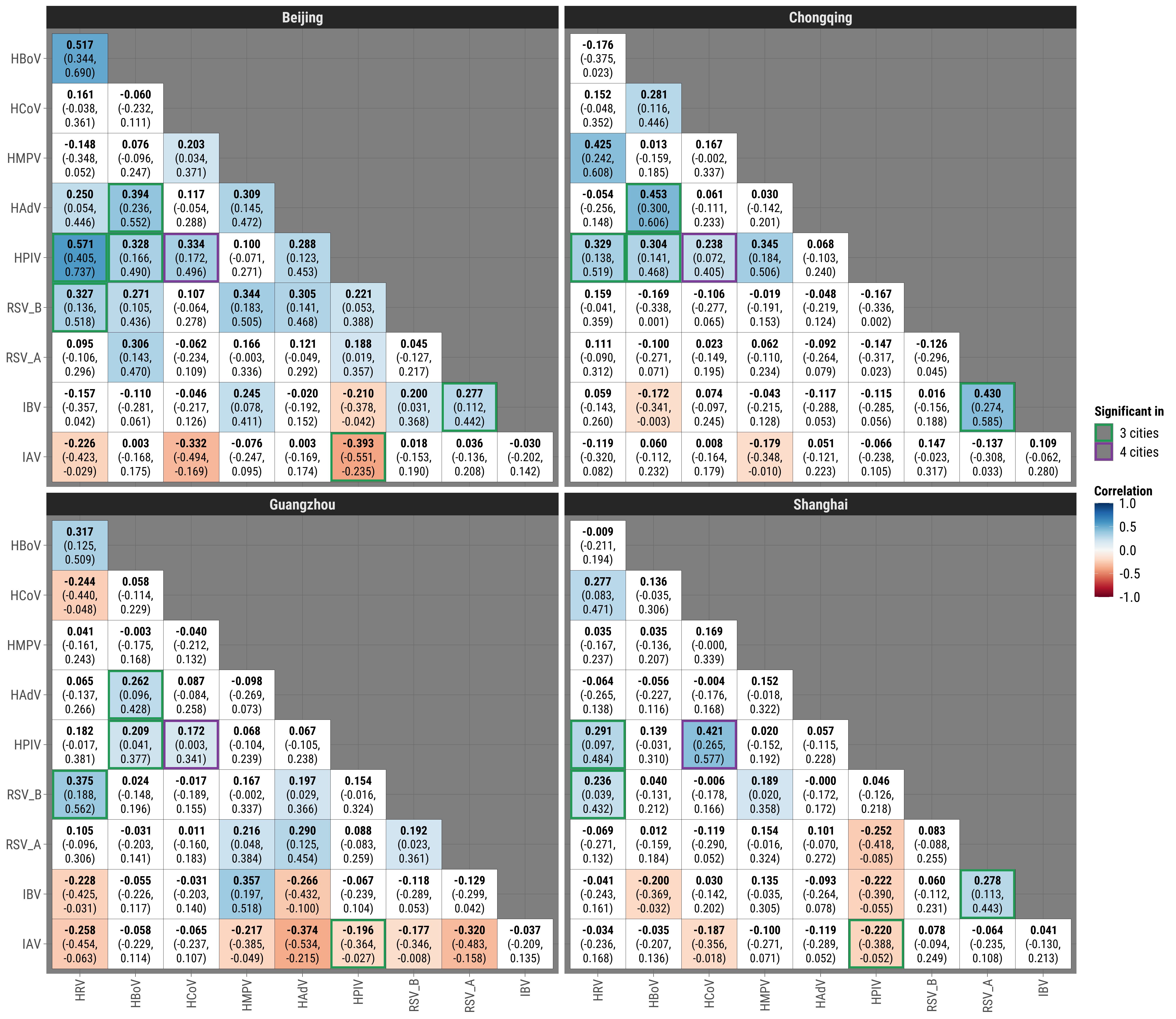


**Figure S9**. Weighted Pearson’s correlation coefficients for the total population studied and 95% confidence intervals of monthly prevalence for each pair of respiratory viruses in four metro cities of China, where weights are the numbers of tests administered. This figure is identical to Figure 3, but with 95% confidence intervals instead of *q-*values. Blue and red indicate statistically significant (*p* < 0.05) positive and negative coefficients, respectively. Green and purple borders indicate virus pairs significant in three and four cities, respectively. The *p*-values are not adjusted for multiple comparisons.


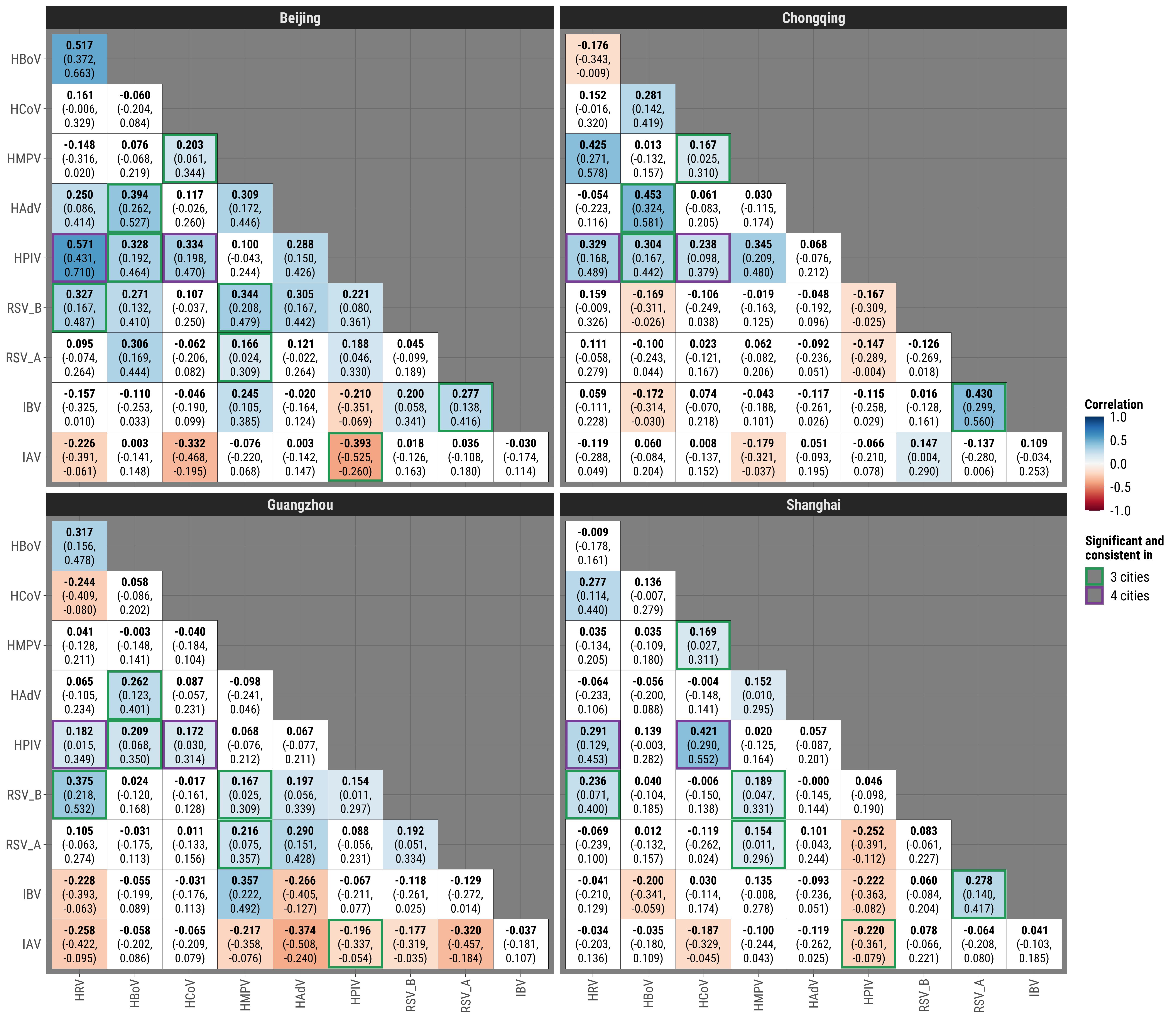


**Figure S10**. Weighted Pearson’s correlation coefficients for the total population studied and 90% confidence intervals of monthly prevalence for each pair of respiratory viruses in four metro cities of China, where weights are the numbers of tests administered. This figure is identical to Figure 3, but with 90% confidence intervals instead of *q-*values. Blue and red indicate statistically significant (*p* < 0.1) positive and negative coefficients, respectively. Green and purple borders indicate virus pairs significant in three and four cities, respectively. The *p*-values are not adjusted for multiple comparisons.

**
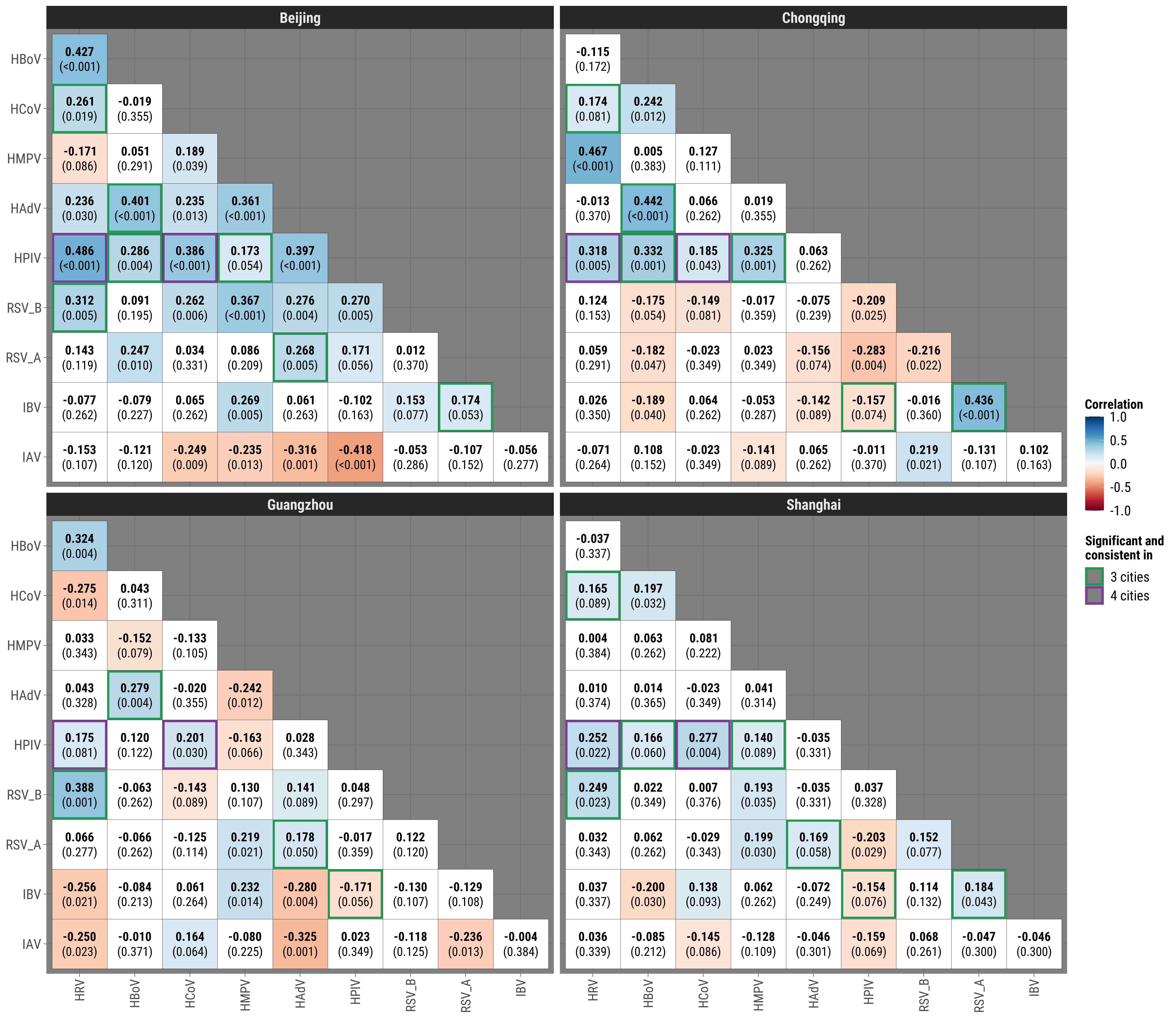
**

**Figure S11**. Weighted Pearson’s correlation coefficients for children <18 years and *q*-values of monthly prevalence for each pair of respiratory viruses in four metro cities of China, where weights are the numbers of tests administered. The *q*-values represent statistical evidence adjusted for multiple comparisons by controlling the false discovery rate. Significant correlations (*q* ≤ 0.10) are shown in colour. Blue and red indicate positive and negative coefficients, respectively. Green and purple borders indicate virus pairs statistically significant with consistent directions in three and four cities, respectively.

**
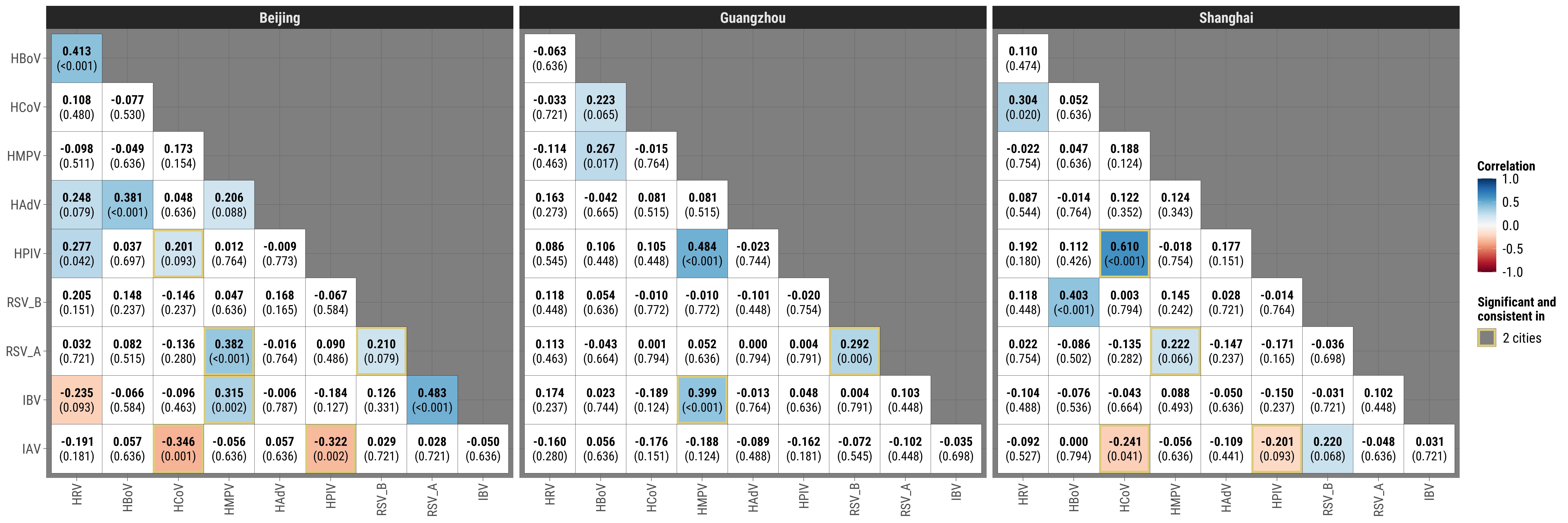
**

**Figure S12**. Weighted Pearson’s correlation coefficients for adults ≥18 years and *q*-values of monthly prevalence for each pair of respiratory viruses in four metro cities of China, where weights are the numbers of tests administered. The *q*-values represent statistical evidence adjusted for multiple comparisons by controlling the false discovery rate. Significant correlations (*q* ≤ 0.10) are shown in colour. Blue and red indicate positive and negative coefficients, respectively. Yellow borders indicate virus pairs statistically significant with consistent directions in two of the three cities, respectively. There was a lower testing rate for adults in Chongqing, therefore that subgroup was not analyzed.


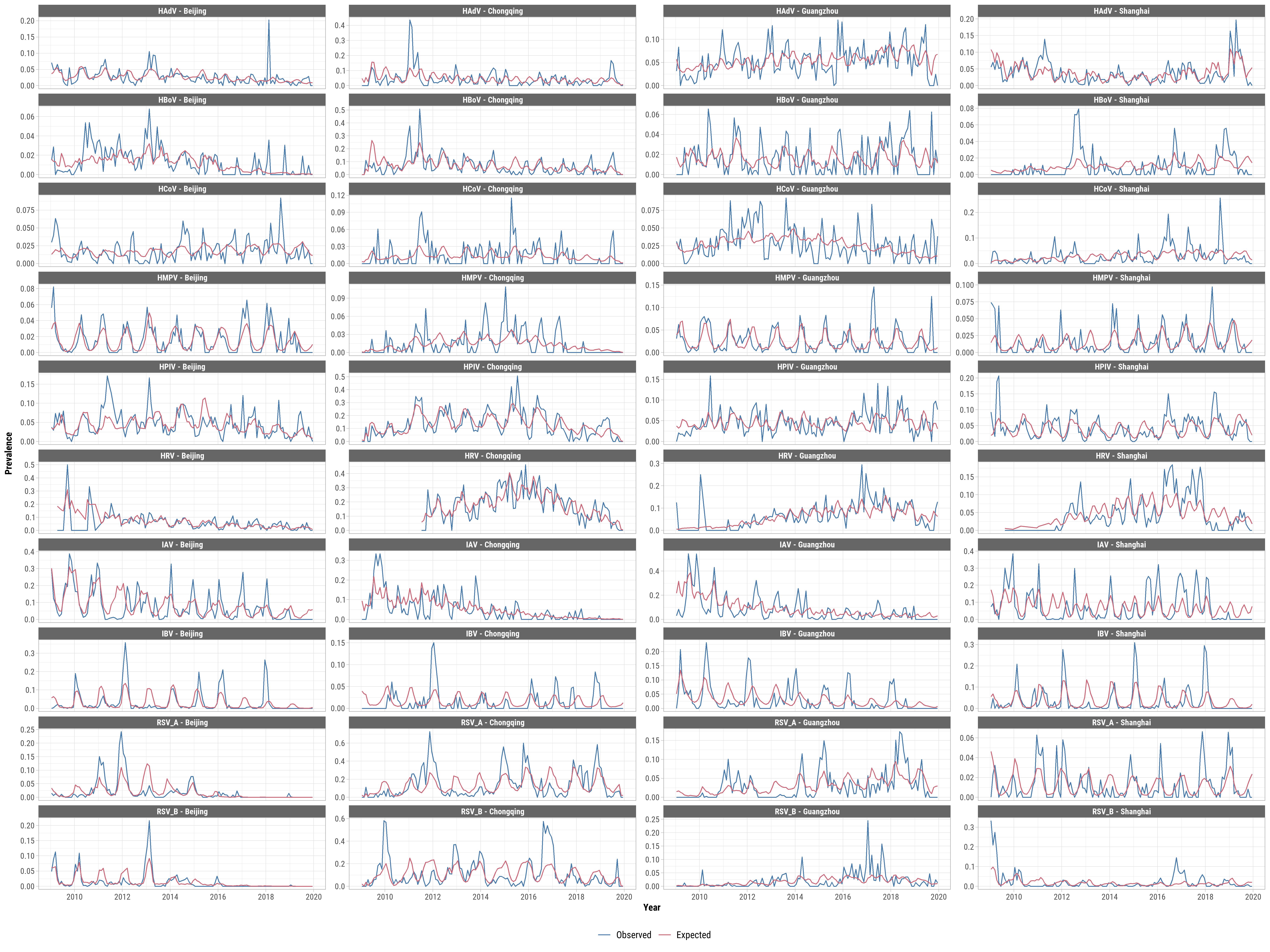


**Figure S13**. The observed and expected prevalence of each virus before applying the multivariate Bayesian hierarchical model, January 2009 to December 2019 in four metro cities, China. The expected prevalence was calculated by fitting generalized linear models for each virus and city with harmonic functions to account for seasonality and polynomials to account for long-term trends, while adjusting for sex and age group (categorized as <5, ≥5-18, ≥18-40, and ≥40 years). Prevalence was the number of infected patients over the total number of patients tested for each virus for each month.


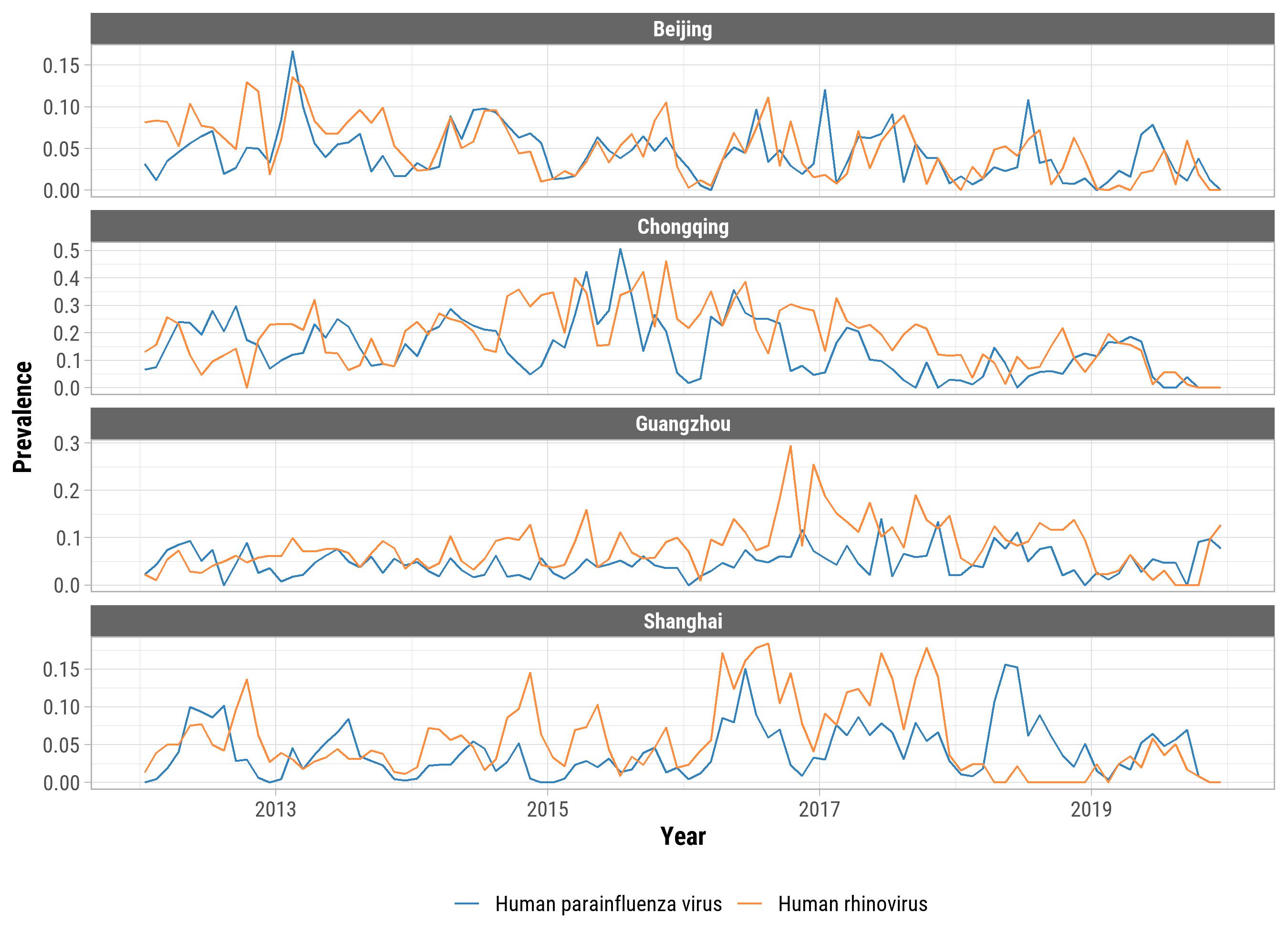


**Figure S14**. Monthly prevalence of human parainfluenza virus and human rhinovirus from January 2009 to December 2019 in four metro cities, China. Prevalence was the number of infected patients over the total number of patients tested for each virus for each month.


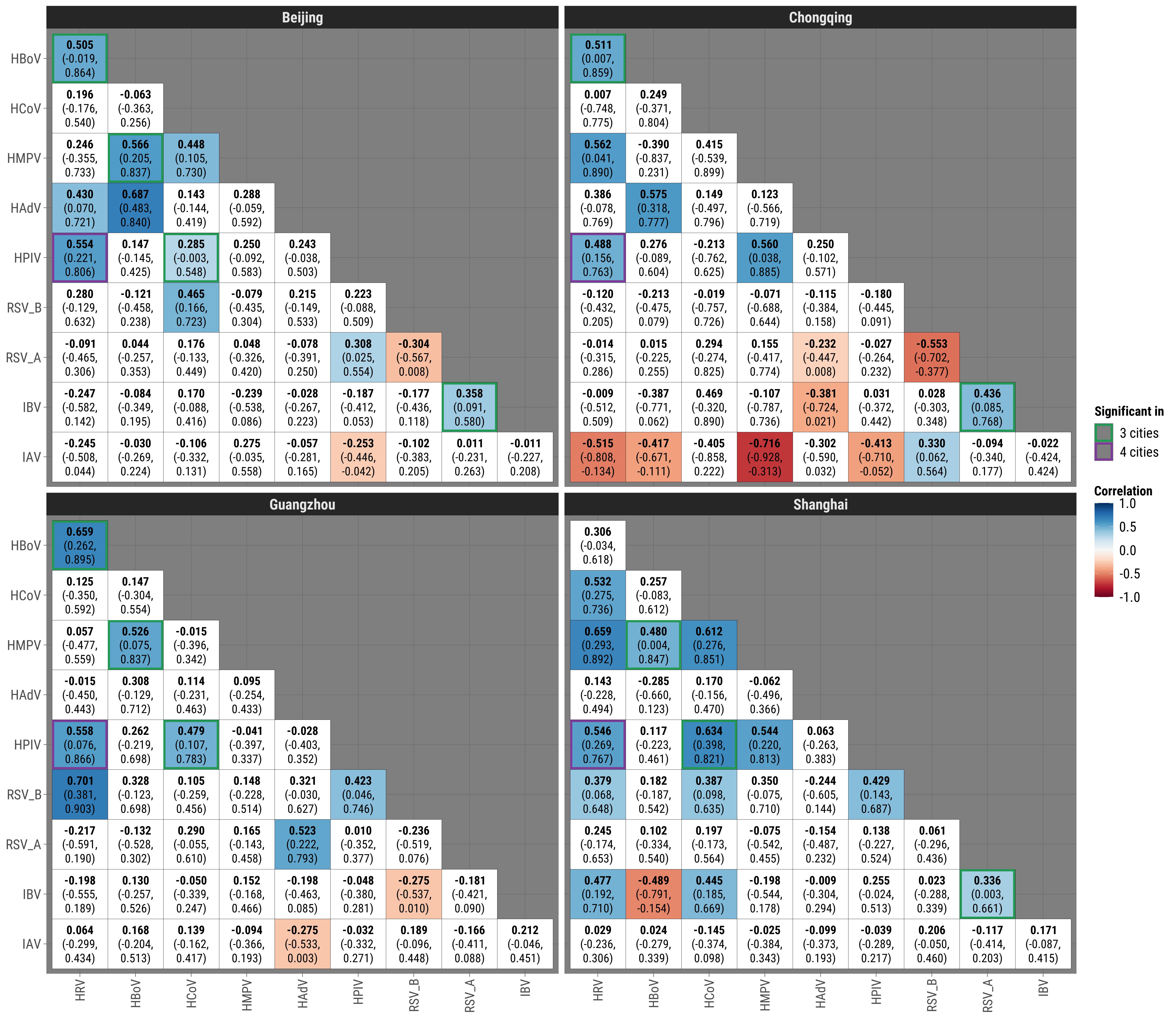


**Figure S15**. Bayesian hierarchical model correlation coefficients for the total population studied and 95% credible intervals, adjusting for age, sex, seasonality, changes in testing frequency, and autocorrelation. This figure is identical to Figure 4, but with 95% credible intervals instead of *q-*values. Blue and red indicate statistically significant (*p* < 0.05) positive and negative coefficients, respectively. Purple borders indicate virus pairs significant in four cities. The *p*-values are not adjusted for multiple comparisons.


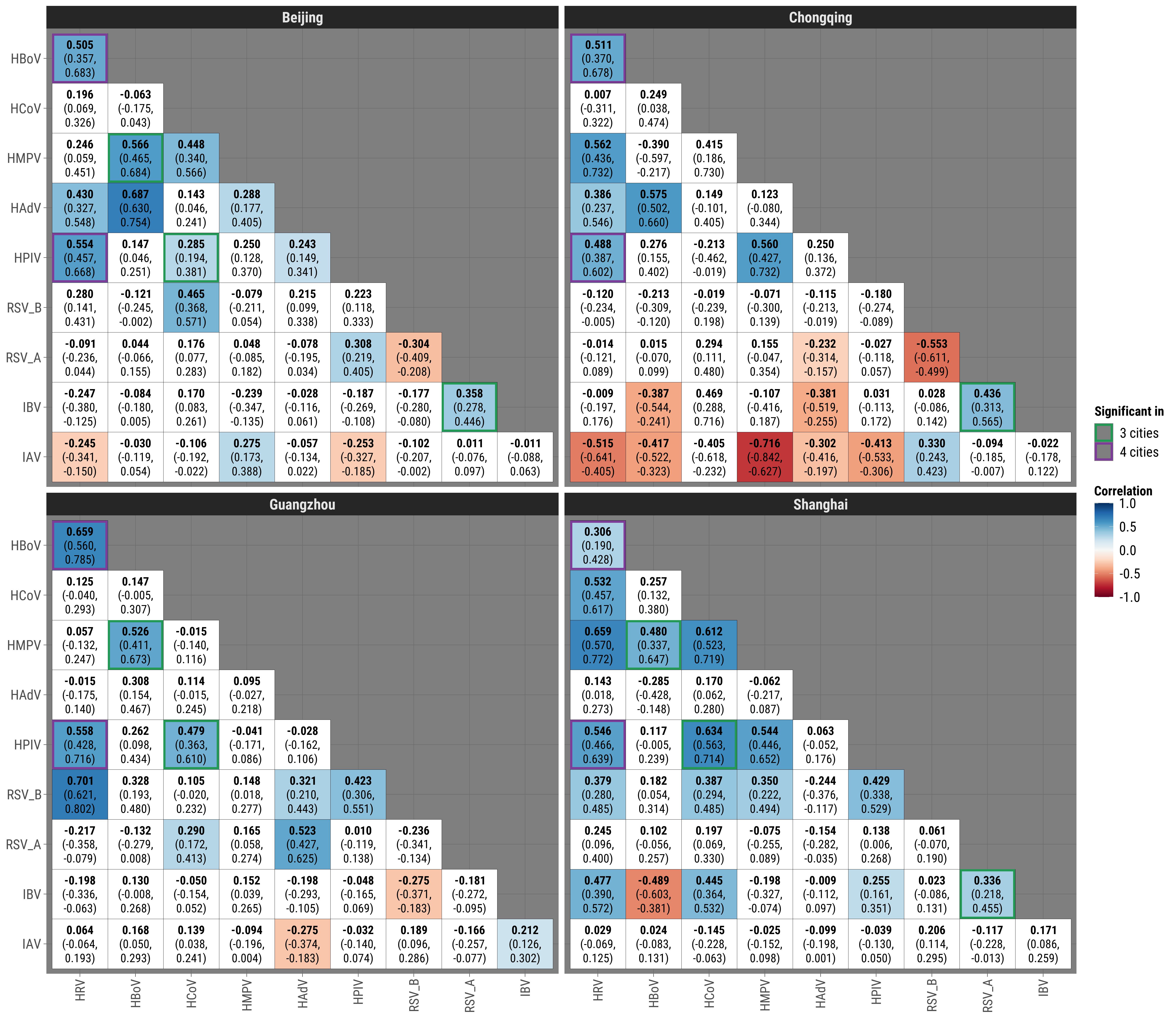


**Figure S16**. Bayesian hierarchical model correlation coefficients for the total population studied and 90% credible intervals, adjusting for age, sex, seasonality, changes in testing frequency, and autocorrelation. This figure is identical to Figure 4, but with 90% credible intervals instead of *q-*values. Blue and red indicate statistically significant (*p* < 0.10) positive and negative coefficients, respectively. Green and purple borders indicate virus pairs significant in three and four cities, respectively. The *p*-values are not adjusted for multiple comparisons.


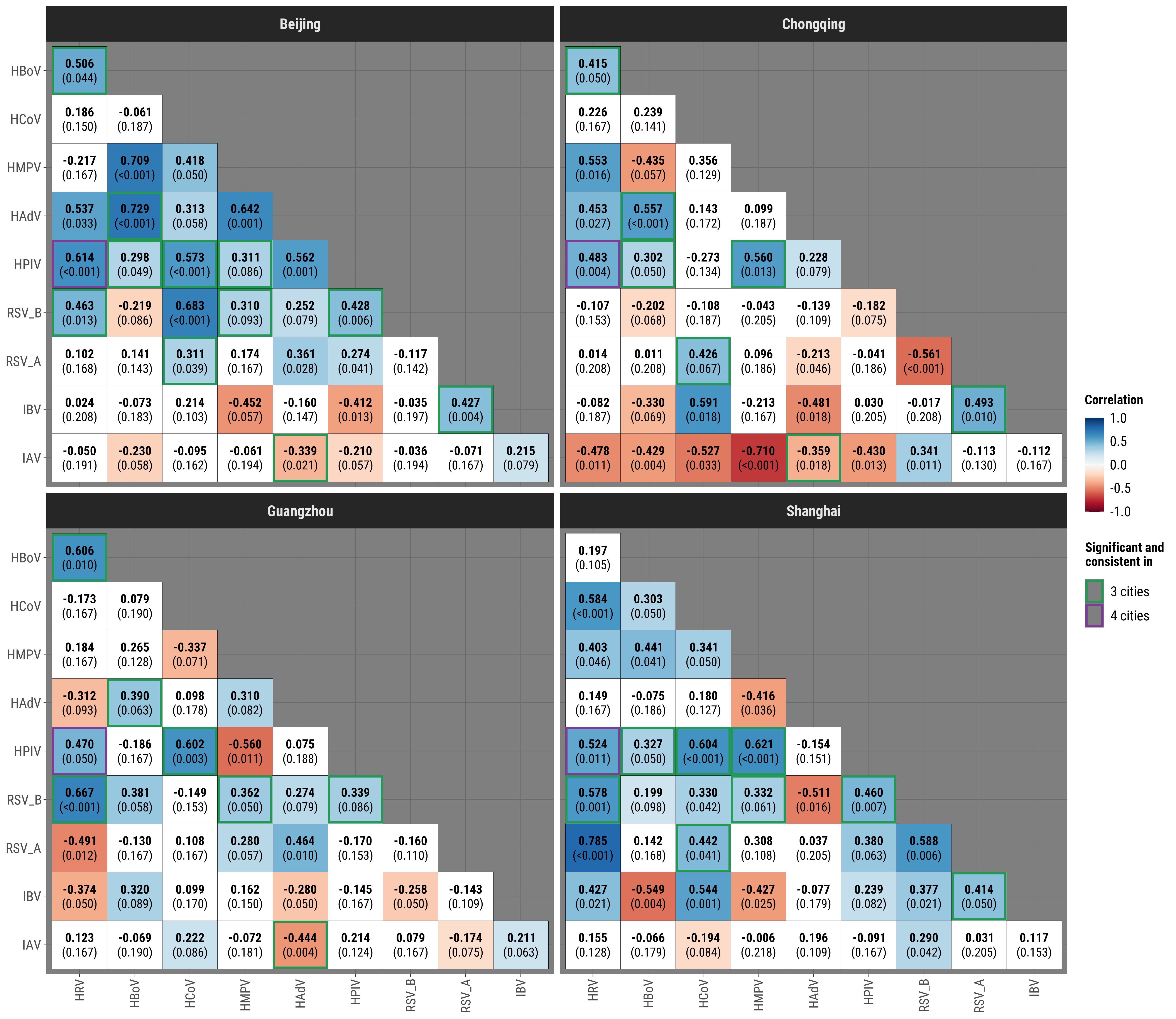


**Figure S17.** Bayesian hierarchical model correlation coefficients for children <18 years and *q*-values, adjusting for age, sex, seasonality, changes in testing frequency, and autocorrelation. The *q*-values represent corrected *p*-values for multiple comparisons by controlling the false discovery rate. Significant correlations (*q* ≤ 0.10) are shown in color. Blue and red indicate positive and negative coefficients, respectively. Green and purple borders indicate virus pairs significant in three and four cities, respectively.


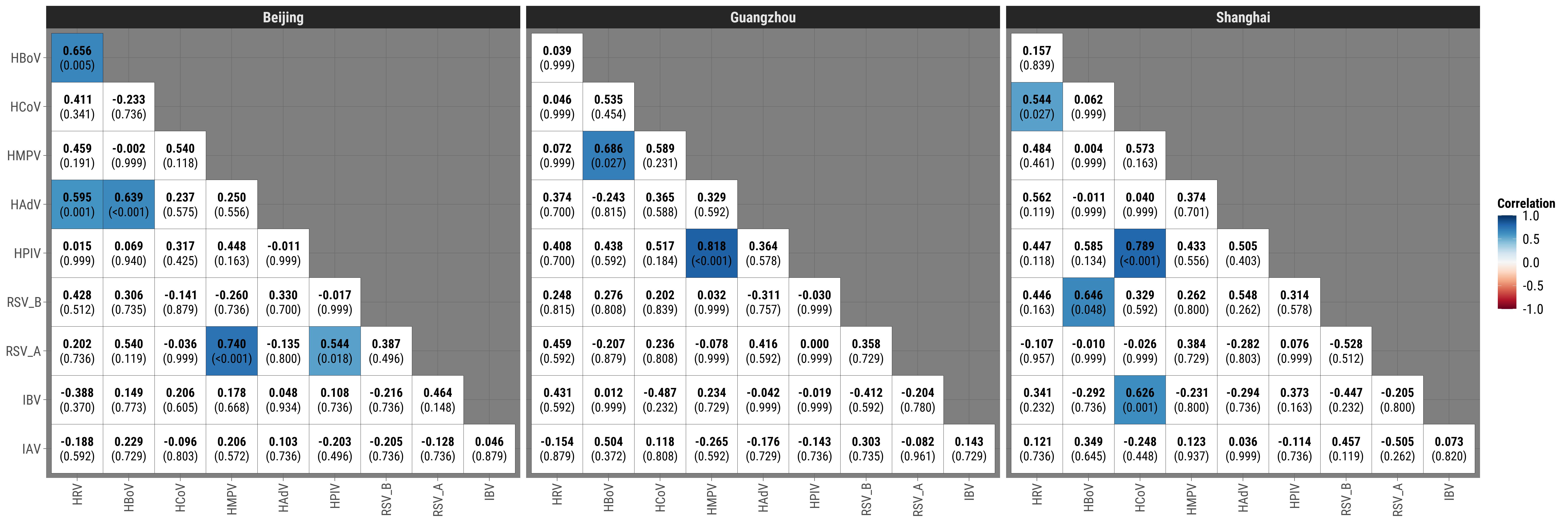


**Figure S18**. Bayesian hierarchical model correlation coefficients for adults ≥18 years and *q*-values, adjusting for age, sex, seasonality, changes in testing frequency, and autocorrelation. The *q*-values represent statistical evidence adjusted for multiple comparisons by controlling the false discovery rate. Significant correlations (*q* ≤ 0.10) are shown in colour. Blue and red indicate positive and negative coefficients, respectively. There was a lower testing rate for adults in Chongqing, therefore that subgroup was excluded from this analysis.

| **Table S1**. Numbers of viruses tested for each patient (N = 101,643). Viruses tested included: influenza A and B; respiratory syncytial virus A and B; and human parainfluenza virus, adenovirus, metapneumovirus, coronavirus, bocavirus, and rhinovirus | | |
| --- | --- | --- |
| Number of viruses tested | Number of patients | Percent |
| 1 | 71 | 0.07 |
| 2 | 4,998 | 4.92 |
| 3 | 451 | 0.44 |
| 4 | 75 | 0.07 |
| 5 | 56 | 0.06 |
| 6 | 63 | 0.06 |
| 7 | 110 | 0.11 |
| 8 | 4,405 | 4.33 |
| 9 | 28,212 | 27.76 |
| 10 | 63,202 | 62.18 |

| **Table S2**. Quasi-Akaike information criteria for harmonic regression models for the total population studied by virus and city. The lowest Quasi-Akaike information criteria for each model is shown in bold. | | | | | | | |
| --- | --- | --- | --- | --- | --- | --- | --- |
| City | Virus | Model 1^a^ | Model 2^b^ | Model 3^c^ | Model 4^d^ | Model 5^e^ | Model 6^f^ |
| Beijing | IAV | 7347.2 | 5901.5 | 5897.9 | 7320.0 | 5813.0 | **5807.4** |
| Beijing | IBV | 3389.4 | 3363.8 | 3187.1 | 3371.1 | 3342.0 | **3172.8** |
| Beijing | HPIV | 3420.1 | 3415.0 | 3298.9 | 3417.3 | 3413.2 | **3298.6** |
| Beijing | RSV_A | 2644.9 | 2629.1 | **2290.1** | 2649.3 | 2634.7 | 2320.1 |
| Beijing | RSV_B | 1704.5 | 1622.5 | 1513.5 | 1698.3 | 1617.2 | **1506.7** |
| Beijing | HAdV | 2942.1 | 2934.3 | **2923.8** | 2942.1 | 2933.1 | 2924.2 |
| Beijing | HMPV | 1716.4 | 1710.0 | **1709.9** | 1720.5 | 1714.3 | 1714.3 |
| Beijing | HCoV | 2145.1 | **2130.4** | 2131.9 | 2150.1 | 2135.6 | 2137.2 |
| Beijing | HBoV | 1637.3 | 1619.7 | **1574.2** | 1638.7 | 1619.9 | 1577.9 |
| Beijing | HRV | 2667.4 | 2472.5 | 2473.8 | 2652.4 | **2456.7** | 2457.9 |
| Shanghai | IAV | 4547.1 | 4407.6 | **4389.0** | 4556.7 | 4414.3 | 4395.1 |
| Shanghai | IBV | 2528.1 | 2495.0 | 2475.9 | 2527.9 | 2493.8 | **2475.5** |
| Shanghai | HPIV | 2366.6 | 2360.2 | **2358.2** | 2372.3 | 2365.8 | 2363.8 |
| Shanghai | RSV_A | **1194.7** | 1194.9 | 1197.7 | 1199.5 | 1199.8 | 1202.6 |
| Shanghai | RSV_B | 1554.5 | 1548.7 | **1533.5** | 1557.6 | 1551.3 | 1536.5 |
| Shanghai | HAdV | 2392.9 | 2370.7 | 2280.6 | 2371.2 | 2350.9 | **2259.4** |
| Shanghai | HMPV | 1309.6 | **1291.7** | 1293.9 | 1312.8 | 1294.4 | 1296.6 |
| Shanghai | HCoV | 2325.9 | 2266.5 | **2249.7** | 2330.7 | 2269.8 | 2252.6 |
| Shanghai | HBoV | 1090.2 | 1055.8 | **1046.3** | 1095.6 | 1061.5 | 1052.1 |
| Shanghai | HRV | 2538.6 | 2542.3 | **2447.7** | 2546.3 | 2550.1 | 2454.9 |
| Guangzhou | IAV | 5111.4 | 4449.1 | 4440.3 | 5083.1 | 4414.8 | **4404.0** |
| Guangzhou | IBV | 2496.7 | 2418.6 | **2417.8** | 2505.8 | 2428.1 | 2427.2 |
| Guangzhou | HPIV | 1998.5 | 1996.3 | **1996.1** | 1999.3 | 1997.1 | 1996.9 |
| Guangzhou | RSV_A | 1610.5 | 1510.3 | **1509.4** | 1612.5 | 1512.4 | 1511.4 |
| Guangzhou | RSV_B | 1285.0 | 1200.7 | **1161.1** | 1291.0 | 1203.8 | 1163.1 |
| Guangzhou | HAdV | 2249.2 | **2232.6** | 2233.1 | 2254.3 | 2237.4 | 2237.8 |
| Guangzhou | HMPV | 1521.9 | 1510.3 | 1510.2 | 1521.3 | 1509.7 | **1509.7** |
| Guangzhou | HCoV | 1807.4 | 1794.0 | **1761.0** | 1808.4 | 1795.4 | 1762.9 |
| Guangzhou | HBoV | 1019.9 | 1015.2 | **1012.4** | 1022.8 | 1018.4 | 1016.0 |
| Guangzhou | HRV | 1957.3 | 1916.1 | 1859.2 | 1957.4 | 1916.2 | **1859.1** |
| Chongqing | IAV | 1637.4 | 1270.3 | 1251.9 | 1624.9 | 1254.3 | **1235.3** |
| Chongqing | IBV | 685.5 | **681.9** | 682.8 | 690.8 | 687.1 | 688.2 |
| Chongqing | HPIV | 1711.1 | 1645.1 | **1544.7** | 1717.4 | 1651.5 | 1549.7 |
| Chongqing | RSV_A | 2135.0 | 2127.7 | 2125.2 | 2134.5 | 2126.9 | **2124.6** |
| Chongqing | RSV_B | 1848.8 | 1808.3 | **1786.2** | 1852.9 | 1812.9 | 1789.9 |
| Chongqing | HAdV | 1278.7 | 1217.5 | **1215.4** | 1284.0 | 1221.8 | 1219.2 |
| Chongqing | HMPV | 602.5 | 599.3 | **564.4** | 607.0 | 603.9 | 568.1 |
| Chongqing | HCoV | 592.7 | 590.2 | **587.0** | 595.9 | 593.5 | 590.0 |
| Chongqing | HBoV | 1367.4 | **1197.0** | 1197.9 | 1370.7 | 1198.4 | 1199.5 |
| Chongqing | HRV | 1452.7 | 1418.4 | 1247.5 | 1454.6 | 1420.2 | **1245.9** |
|  | | | | | | | |
|  | | | | | | | |
| ^a^ Model 1: $\log\left( \frac{\pi_{ymiv}}{1-\pi_{ymiv}} \right)=\beta_{v0}+\beta_{v1}{SEX}_{ymi}+{\boldsymbol{\beta}_{\boldsymbol{v}\boldsymbol{2}}}^{\boldsymbol{'}}\boldsymbol{AGE}_{\boldsymbol{ymi}}+\beta_{v3}sin\left( \frac{2\pi m}{12} \right)+\beta_{v4}cos\left( \frac{2\pi m}{12} \right)+\beta_{v5} sin\left( \frac{2^{2}\pi m}{12} \right)+\beta_{v6}cos\left( \frac{2^{2}\pi m}{12} \right)$ | | | | | | | |
| ^b^ Model 2: $\log\left( \frac{\pi_{ymiv}}{1-\pi_{ymiv}} \right)=\mathrm{Model} 1+\beta_{v7}y$ | | | | | | | |
| ^c^ Model 3: $\log\left( \frac{\pi_{ymiv}}{1-\pi_{ymiv}} \right)=\mathrm{Model} 1+\beta_{v7}y+\beta_{v8}y^{2}$ | | | | | | | |
| ^d^ Model 4: $\log\left( \frac{\pi_{ymiv}}{1-\pi_{ymiv}} \right)=\mathrm{Model} 1+\beta_{v7} sin\left( \frac{2^{3}\pi m}{12} \right)+\beta_{v8}cos\left( \frac{2^{3}\pi m}{12} \right)$ | | | | | | | |
| ^e^ Model 5: $\log\left( \frac{\pi_{ymiv}}{1-\pi_{ymiv}} \right)=\mathrm{Model} 1+\beta_{v7} sin\left( \frac{2^{3}\pi m}{12} \right)+\beta_{v8}cos\left( \frac{2^{3}\pi m}{12} \right)+\beta_{v9}y$ | | | | | | | |
| ^f^ Model 6: $\log\left( \frac{\pi_{ymiv}}{1-\pi_{ymiv}} \right)=\mathrm{Model}1+\beta_{v7} sin\left( \frac{2^{3}\pi m}{12} \right)+\beta_{v8}cos\left( \frac{2^{3}\pi m}{12} \right)+\beta_{v9}y+\beta_{v10}y^{2}$ | | | | | | | |

| **Table S3**. Mean, standard deviation, and quantiles of the marginal posterior distribution for ρ, and convergence diagnostics for Bayesian hierarchical model. | | | | | | | | | | | | | |
| --- | --- | --- | --- | --- | --- | --- | --- | --- | --- | --- | --- | --- | --- |
| City | Virus 1 | Virus 2 | mu.vect | sd.vect | 2.5% | 5.0% | 50.0% | 95.0% | 97.5% | Rhat | n.eff | pD | DIC |
| Beijing | IBV | IAV | -0.011 | 0.111 | -0.227 | -0.088 | -0.012 | 0.063 | 0.208 | 1.002 | 2500 | 264.9 | 1322.5 |
| Beijing | RSV_A | IAV | 0.011 | 0.127 | -0.231 | -0.076 | 0.009 | 0.097 | 0.263 | 1.003 | 1700 | 253.8 | 1282.4 |
| Beijing | RSV_B | IAV | -0.102 | 0.150 | -0.383 | -0.207 | -0.107 | -0.002 | 0.205 | 1.004 | 1300 | 249.2 | 1218.0 |
| Beijing | HPIV | IAV | -0.253 | 0.104 | -0.446 | -0.327 | -0.257 | -0.185 | -0.042 | 1.001 | 6000 | 266.1 | 1544.8 |
| Beijing | HADV | IAV | -0.057 | 0.114 | -0.281 | -0.134 | -0.059 | 0.022 | 0.165 | 1.003 | 1900 | 274.2 | 1470.9 |
| Beijing | HMPV | IAV | 0.275 | 0.154 | -0.035 | 0.173 | 0.278 | 0.388 | 0.558 | 1.006 | 930 | 270.9 | 1367.3 |
| Beijing | HCOV | IAV | -0.106 | 0.120 | -0.332 | -0.192 | -0.108 | -0.022 | 0.131 | 1.002 | 3200 | 278.1 | 1429.8 |
| Beijing | HBOV | IAV | -0.030 | 0.125 | -0.269 | -0.119 | -0.032 | 0.054 | 0.224 | 1.001 | 6000 | 285.5 | 1359.3 |
| Beijing | HRV | IAV | -0.245 | 0.140 | -0.508 | -0.341 | -0.250 | -0.150 | 0.044 | 1.002 | 2800 | 223.3 | 1117.2 |
| Beijing | RSV_A | IBV | 0.358 | 0.126 | 0.091 | 0.278 | 0.364 | 0.446 | 0.580 | 1.002 | 3300 | 211.9 | 1038.0 |
| Beijing | RSV_B | IBV | -0.177 | 0.143 | -0.436 | -0.280 | -0.184 | -0.080 | 0.118 | 1.002 | 3000 | 214.4 | 984.3 |
| Beijing | HPIV | IBV | -0.187 | 0.120 | -0.412 | -0.269 | -0.191 | -0.108 | 0.053 | 1.001 | 6000 | 252.1 | 1331.3 |
| Beijing | HADV | IBV | -0.028 | 0.127 | -0.267 | -0.116 | -0.029 | 0.061 | 0.223 | 1.002 | 2400 | 235.2 | 1232.4 |
| Beijing | HMPV | IBV | -0.239 | 0.159 | -0.538 | -0.347 | -0.243 | -0.135 | 0.086 | 1.004 | 1600 | 251.2 | 1151.0 |
| Beijing | HCOV | IBV | 0.170 | 0.130 | -0.088 | 0.083 | 0.172 | 0.261 | 0.416 | 1.003 | 1800 | 248.5 | 1198.9 |
| Beijing | HBOV | IBV | -0.084 | 0.138 | -0.349 | -0.180 | -0.084 | 0.005 | 0.195 | 1.005 | 1200 | 253.6 | 1128.3 |
| Beijing | HRV | IBV | -0.247 | 0.186 | -0.582 | -0.380 | -0.258 | -0.125 | 0.142 | 1.005 | 1200 | 182.0 | 917.3 |
| Beijing | RSV_B | RSV_A | -0.304 | 0.148 | -0.567 | -0.409 | -0.313 | -0.208 | 0.008 | 1.001 | 6000 | 211.1 | 953.0 |
| Beijing | HPIV | RSV_A | 0.308 | 0.136 | 0.025 | 0.219 | 0.317 | 0.405 | 0.554 | 1.001 | 5000 | 240.7 | 1292.2 |
| Beijing | HADV | RSV_A | -0.078 | 0.165 | -0.391 | -0.195 | -0.083 | 0.034 | 0.250 | 1.002 | 3400 | 213.0 | 1180.3 |
| Beijing | HMPV | RSV_A | 0.048 | 0.193 | -0.326 | -0.085 | 0.048 | 0.182 | 0.420 | 1.001 | 6000 | 234.1 | 1104.3 |
| Beijing | HCOV | RSV_A | 0.176 | 0.150 | -0.133 | 0.077 | 0.181 | 0.283 | 0.449 | 1.004 | 1400 | 233.9 | 1155.5 |
| Beijing | HBOV | RSV_A | 0.044 | 0.159 | -0.257 | -0.066 | 0.040 | 0.155 | 0.353 | 1.001 | 4500 | 241.0 | 1084.8 |
| Beijing | HRV | RSV_A | -0.091 | 0.201 | -0.465 | -0.236 | -0.094 | 0.044 | 0.306 | 1.003 | 1800 | 174.8 | 897.1 |
| Beijing | HPIV | RSV_B | 0.223 | 0.155 | -0.088 | 0.118 | 0.228 | 0.333 | 0.509 | 1.002 | 2600 | 226.1 | 1215.5 |
| Beijing | HADV | RSV_B | 0.215 | 0.176 | -0.149 | 0.099 | 0.224 | 0.338 | 0.533 | 1.003 | 2000 | 216.7 | 1123.6 |
| Beijing | HMPV | RSV_B | -0.079 | 0.191 | -0.435 | -0.211 | -0.086 | 0.054 | 0.304 | 1.003 | 1700 | 227.3 | 1036.9 |
| Beijing | HCOV | RSV_B | 0.465 | 0.146 | 0.166 | 0.368 | 0.472 | 0.571 | 0.723 | 1.007 | 880 | 236.7 | 1104.2 |
| Beijing | HBOV | RSV_B | -0.121 | 0.177 | -0.458 | -0.245 | -0.125 | -0.002 | 0.238 | 1.006 | 920 | 236.6 | 1021.7 |
| Beijing | HRV | RSV_B | 0.280 | 0.202 | -0.129 | 0.141 | 0.290 | 0.431 | 0.632 | 1.002 | 2500 | 172.7 | 836.0 |
| Beijing | HADV | HPIV | 0.243 | 0.139 | -0.038 | 0.149 | 0.248 | 0.341 | 0.503 | 1.003 | 2300 | 251.2 | 1470.2 |
| Beijing | HMPV | HPIV | 0.250 | 0.175 | -0.092 | 0.128 | 0.253 | 0.370 | 0.583 | 1.004 | 1400 | 259.1 | 1381.6 |
| Beijing | HCOV | HPIV | 0.285 | 0.141 | -0.003 | 0.194 | 0.292 | 0.381 | 0.548 | 1.002 | 2500 | 268.4 | 1442.3 |
| Beijing | HBOV | HPIV | 0.147 | 0.148 | -0.145 | 0.046 | 0.152 | 0.251 | 0.425 | 1.003 | 1700 | 271.9 | 1366.7 |
| Beijing | HRV | HPIV | 0.554 | 0.152 | 0.221 | 0.457 | 0.570 | 0.668 | 0.806 | 1.005 | 1200 | 190.4 | 1120.8 |
| Beijing | HMPV | HADV | 0.288 | 0.167 | -0.059 | 0.177 | 0.295 | 0.405 | 0.592 | 1.001 | 6000 | 239.5 | 1278.6 |
| Beijing | HCOV | HADV | 0.143 | 0.143 | -0.144 | 0.046 | 0.145 | 0.241 | 0.419 | 1.002 | 3600 | 252.5 | 1343.6 |
| Beijing | HBOV | HADV | 0.687 | 0.093 | 0.483 | 0.630 | 0.697 | 0.754 | 0.840 | 1.004 | 1500 | 223.6 | 1228.4 |
| Beijing | HRV | HADV | 0.430 | 0.166 | 0.070 | 0.327 | 0.442 | 0.548 | 0.721 | 1.005 | 1200 | 180.5 | 1036.4 |
| Beijing | HCOV | HMPV | 0.448 | 0.162 | 0.105 | 0.340 | 0.459 | 0.566 | 0.730 | 1.004 | 1500 | 251.4 | 1242.5 |
| Beijing | HBOV | HMPV | 0.566 | 0.164 | 0.205 | 0.465 | 0.581 | 0.684 | 0.837 | 1.013 | 500 | 248.4 | 1169.5 |
| Beijing | HRV | HMPV | 0.246 | 0.281 | -0.355 | 0.059 | 0.266 | 0.451 | 0.733 | 1.009 | 660 | 195.3 | 1002.1 |
| Beijing | HBOV | HCOV | -0.063 | 0.159 | -0.363 | -0.175 | -0.067 | 0.043 | 0.256 | 1.002 | 3200 | 265.6 | 1233.7 |
| Beijing | HRV | HCOV | 0.196 | 0.186 | -0.176 | 0.069 | 0.201 | 0.326 | 0.540 | 1.002 | 2500 | 197.5 | 1035.0 |
| Beijing | HRV | HBOV | 0.505 | 0.233 | -0.019 | 0.357 | 0.534 | 0.683 | 0.864 | 1.028 | 210 | 208.4 | 991.0 |
| Shanghai | IBV | IAV | 0.171 | 0.127 | -0.087 | 0.086 | 0.173 | 0.259 | 0.415 | 1.004 | 1400 | 252.0 | 1156.9 |
| Shanghai | RSV_A | IAV | -0.117 | 0.157 | -0.414 | -0.228 | -0.122 | -0.013 | 0.203 | 1.003 | 1800 | 263.6 | 1141.7 |
| Shanghai | RSV_B | IAV | 0.206 | 0.131 | -0.050 | 0.114 | 0.208 | 0.295 | 0.460 | 1.003 | 2200 | 273.5 | 1142.6 |
| Shanghai | HPIV | IAV | -0.039 | 0.131 | -0.289 | -0.130 | -0.041 | 0.050 | 0.217 | 1.002 | 3500 | 289.6 | 1393.5 |
| Shanghai | HADV | IAV | -0.099 | 0.146 | -0.373 | -0.198 | -0.099 | 0.001 | 0.193 | 1.003 | 2300 | 272.5 | 1363.6 |
| Shanghai | HMPV | IAV | -0.025 | 0.186 | -0.384 | -0.152 | -0.026 | 0.098 | 0.343 | 1.003 | 3100 | 292.6 | 1222.9 |
| Shanghai | HCOV | IAV | -0.145 | 0.121 | -0.374 | -0.228 | -0.148 | -0.063 | 0.098 | 1.001 | 6000 | 260.9 | 1275.7 |
| Shanghai | HBOV | IAV | 0.024 | 0.158 | -0.279 | -0.083 | 0.021 | 0.131 | 0.339 | 1.006 | 880 | 266.8 | 1086.9 |
| Shanghai | HRV | IAV | 0.029 | 0.140 | -0.236 | -0.069 | 0.029 | 0.125 | 0.306 | 1.002 | 3500 | 216.5 | 1020.9 |
| Shanghai | RSV_A | IBV | 0.336 | 0.170 | 0.003 | 0.218 | 0.340 | 0.455 | 0.661 | 1.012 | 460 | 236.5 | 988.0 |
| Shanghai | RSV_B | IBV | 0.023 | 0.160 | -0.288 | -0.086 | 0.024 | 0.131 | 0.339 | 1.004 | 1400 | 260.3 | 1006.4 |
| Shanghai | HPIV | IBV | 0.255 | 0.138 | -0.024 | 0.161 | 0.259 | 0.351 | 0.513 | 1.002 | 2600 | 259.3 | 1239.8 |
| Shanghai | HADV | IBV | -0.009 | 0.154 | -0.304 | -0.112 | -0.009 | 0.097 | 0.294 | 1.003 | 1700 | 260.7 | 1227.7 |
| Shanghai | HMPV | IBV | -0.198 | 0.186 | -0.544 | -0.327 | -0.201 | -0.074 | 0.178 | 1.005 | 1100 | 286.0 | 1093.4 |
| Shanghai | HCOV | IBV | 0.445 | 0.124 | 0.185 | 0.364 | 0.450 | 0.532 | 0.669 | 1.004 | 1500 | 256.2 | 1148.8 |
| Shanghai | HBOV | IBV | -0.489 | 0.164 | -0.791 | -0.603 | -0.496 | -0.381 | -0.154 | 1.020 | 510 | 252.4 | 954.2 |
| Shanghai | HRV | IBV | 0.477 | 0.134 | 0.192 | 0.390 | 0.488 | 0.572 | 0.710 | 1.003 | 2000 | 197.2 | 895.8 |
| Shanghai | RSV_B | RSV_A | 0.061 | 0.188 | -0.296 | -0.070 | 0.058 | 0.190 | 0.436 | 1.002 | 3400 | 263.7 | 980.6 |
| Shanghai | HPIV | RSV_A | 0.138 | 0.191 | -0.227 | 0.006 | 0.133 | 0.268 | 0.524 | 1.009 | 650 | 261.4 | 1212.4 |
| Shanghai | HADV | RSV_A | -0.154 | 0.184 | -0.487 | -0.282 | -0.164 | -0.035 | 0.232 | 1.003 | 2000 | 258.9 | 1195.4 |
| Shanghai | HMPV | RSV_A | -0.075 | 0.254 | -0.542 | -0.255 | -0.087 | 0.089 | 0.455 | 1.011 | 550 | 289.8 | 1068.6 |
| Shanghai | HCOV | RSV_A | 0.197 | 0.189 | -0.173 | 0.069 | 0.196 | 0.330 | 0.564 | 1.004 | 1400 | 248.0 | 1111.0 |
| Shanghai | HBOV | RSV_A | 0.102 | 0.226 | -0.334 | -0.056 | 0.099 | 0.257 | 0.540 | 1.005 | 1200 | 252.4 | 920.7 |
| Shanghai | HRV | RSV_A | 0.245 | 0.216 | -0.174 | 0.096 | 0.247 | 0.400 | 0.653 | 1.006 | 970 | 202.6 | 907.3 |
| Shanghai | HPIV | RSV_B | 0.429 | 0.139 | 0.143 | 0.338 | 0.431 | 0.529 | 0.687 | 1.004 | 1600 | 258.9 | 1203.0 |
| Shanghai | HADV | RSV_B | -0.244 | 0.191 | -0.605 | -0.376 | -0.249 | -0.117 | 0.144 | 1.010 | 610 | 281.4 | 1217.3 |
| Shanghai | HMPV | RSV_B | 0.350 | 0.200 | -0.075 | 0.222 | 0.361 | 0.494 | 0.710 | 1.006 | 990 | 279.7 | 1047.9 |
| Shanghai | HCOV | RSV_B | 0.387 | 0.139 | 0.098 | 0.294 | 0.394 | 0.485 | 0.635 | 1.002 | 2800 | 266.2 | 1118.3 |
| Shanghai | HBOV | RSV_B | 0.182 | 0.188 | -0.187 | 0.054 | 0.184 | 0.314 | 0.542 | 1.003 | 1700 | 266.7 | 928.6 |
| Shanghai | HRV | RSV_B | 0.379 | 0.148 | 0.068 | 0.280 | 0.385 | 0.485 | 0.648 | 1.001 | 6000 | 195.4 | 887.8 |
| Shanghai | HADV | HPIV | 0.063 | 0.166 | -0.263 | -0.052 | 0.062 | 0.176 | 0.383 | 1.004 | 1500 | 288.0 | 1453.0 |
| Shanghai | HMPV | HPIV | 0.544 | 0.153 | 0.220 | 0.446 | 0.555 | 0.652 | 0.813 | 1.005 | 1200 | 284.2 | 1280.2 |
| Shanghai | HCOV | HPIV | 0.634 | 0.109 | 0.398 | 0.563 | 0.644 | 0.714 | 0.821 | 1.004 | 1500 | 278.7 | 1376.6 |
| Shanghai | HBOV | HPIV | 0.117 | 0.177 | -0.223 | -0.005 | 0.115 | 0.239 | 0.461 | 1.004 | 1500 | 271.9 | 1167.1 |
| Shanghai | HRV | HPIV | 0.546 | 0.129 | 0.269 | 0.466 | 0.555 | 0.639 | 0.767 | 1.004 | 1500 | 203.6 | 1056.8 |
| Shanghai | HMPV | HADV | -0.062 | 0.223 | -0.496 | -0.217 | -0.061 | 0.087 | 0.366 | 1.010 | 590 | 295.1 | 1287.5 |
| Shanghai | HCOV | HADV | 0.170 | 0.160 | -0.156 | 0.062 | 0.173 | 0.280 | 0.470 | 1.002 | 3200 | 270.7 | 1347.3 |
| Shanghai | HBOV | HADV | -0.285 | 0.202 | -0.660 | -0.428 | -0.293 | -0.148 | 0.123 | 1.013 | 440 | 267.9 | 1150.0 |
| Shanghai | HRV | HADV | 0.143 | 0.184 | -0.228 | 0.018 | 0.145 | 0.273 | 0.494 | 1.001 | 5000 | 223.1 | 1065.5 |
| Shanghai | HCOV | HMPV | 0.612 | 0.149 | 0.276 | 0.523 | 0.630 | 0.719 | 0.851 | 1.008 | 740 | 276.5 | 1184.2 |
| Shanghai | HBOV | HMPV | 0.480 | 0.220 | 0.004 | 0.337 | 0.500 | 0.647 | 0.847 | 1.009 | 730 | 286.0 | 1004.0 |
| Shanghai | HRV | HMPV | 0.659 | 0.155 | 0.293 | 0.570 | 0.684 | 0.772 | 0.892 | 1.008 | 680 | 201.2 | 953.9 |
| Shanghai | HBOV | HCOV | 0.257 | 0.179 | -0.083 | 0.132 | 0.255 | 0.380 | 0.612 | 1.002 | 3000 | 257.9 | 1063.1 |
| Shanghai | HRV | HCOV | 0.532 | 0.119 | 0.275 | 0.457 | 0.544 | 0.617 | 0.736 | 1.004 | 1600 | 194.9 | 1012.6 |
| Shanghai | HRV | HBOV | 0.306 | 0.170 | -0.034 | 0.190 | 0.312 | 0.428 | 0.618 | 1.006 | 950 | 213.4 | 912.8 |
| Guangzhou | IBV | IAV | 0.212 | 0.126 | -0.046 | 0.126 | 0.215 | 0.302 | 0.451 | 1.002 | 2400 | 264.2 | 1231.2 |
| Guangzhou | RSV_A | IAV | -0.166 | 0.129 | -0.411 | -0.257 | -0.169 | -0.077 | 0.088 | 1.003 | 2000 | 279.2 | 1254.3 |
| Guangzhou | RSV_B | IAV | 0.189 | 0.140 | -0.096 | 0.096 | 0.194 | 0.286 | 0.448 | 1.003 | 2300 | 253.1 | 1192.2 |
| Guangzhou | HPIV | IAV | -0.032 | 0.154 | -0.332 | -0.140 | -0.033 | 0.074 | 0.271 | 1.003 | 2300 | 259.5 | 1419.8 |
| Guangzhou | HADV | IAV | -0.275 | 0.138 | -0.533 | -0.374 | -0.281 | -0.183 | 0.003 | 1.006 | 940 | 280.6 | 1453.7 |
| Guangzhou | HMPV | IAV | -0.094 | 0.144 | -0.366 | -0.196 | -0.097 | 0.004 | 0.193 | 1.002 | 3300 | 269.7 | 1309.1 |
| Guangzhou | HCOV | IAV | 0.139 | 0.149 | -0.162 | 0.038 | 0.143 | 0.241 | 0.417 | 1.003 | 2000 | 270.2 | 1350.6 |
| Guangzhou | HBOV | IAV | 0.168 | 0.182 | -0.204 | 0.050 | 0.169 | 0.293 | 0.513 | 1.007 | 860 | 293.9 | 1267.5 |
| Guangzhou | HRV | IAV | 0.064 | 0.189 | -0.299 | -0.064 | 0.061 | 0.193 | 0.434 | 1.003 | 2300 | 229.4 | 1051.2 |
| Guangzhou | RSV_A | IBV | -0.181 | 0.131 | -0.421 | -0.272 | -0.186 | -0.095 | 0.090 | 1.004 | 1500 | 259.5 | 1080.0 |
| Guangzhou | RSV_B | IBV | -0.275 | 0.140 | -0.537 | -0.371 | -0.279 | -0.183 | 0.010 | 1.006 | 970 | 237.2 | 1020.5 |
| Guangzhou | HPIV | IBV | -0.048 | 0.169 | -0.380 | -0.165 | -0.048 | 0.069 | 0.281 | 1.001 | 6000 | 251.2 | 1257.0 |
| Guangzhou | HADV | IBV | -0.198 | 0.140 | -0.463 | -0.293 | -0.202 | -0.105 | 0.085 | 1.001 | 6000 | 258.4 | 1278.1 |
| Guangzhou | HMPV | IBV | 0.152 | 0.163 | -0.168 | 0.039 | 0.154 | 0.265 | 0.466 | 1.005 | 1200 | 243.6 | 1128.0 |
| Guangzhou | HCOV | IBV | -0.050 | 0.152 | -0.339 | -0.154 | -0.051 | 0.052 | 0.247 | 1.003 | 2400 | 250.1 | 1175.6 |
| Guangzhou | HBOV | IBV | 0.130 | 0.202 | -0.257 | -0.008 | 0.127 | 0.268 | 0.526 | 1.004 | 1400 | 286.1 | 1108.0 |
| Guangzhou | HRV | IBV | -0.198 | 0.195 | -0.555 | -0.336 | -0.203 | -0.063 | 0.189 | 1.006 | 980 | 200.1 | 922.2 |
| Guangzhou | RSV_B | RSV_A | -0.236 | 0.152 | -0.519 | -0.341 | -0.241 | -0.134 | 0.076 | 1.004 | 1600 | 249.7 | 1037.2 |
| Guangzhou | HPIV | RSV_A | 0.010 | 0.188 | -0.352 | -0.119 | 0.013 | 0.138 | 0.377 | 1.003 | 1700 | 255.4 | 1264.4 |
| Guangzhou | HADV | RSV_A | 0.523 | 0.145 | 0.222 | 0.427 | 0.529 | 0.625 | 0.793 | 1.011 | 570 | 271.4 | 1296.3 |
| Guangzhou | HMPV | RSV_A | 0.165 | 0.156 | -0.143 | 0.058 | 0.167 | 0.274 | 0.458 | 1.003 | 2100 | 251.8 | 1138.7 |
| Guangzhou | HCOV | RSV_A | 0.290 | 0.172 | -0.055 | 0.172 | 0.296 | 0.413 | 0.610 | 1.008 | 690 | 251.8 | 1181.8 |
| Guangzhou | HBOV | RSV_A | -0.132 | 0.211 | -0.528 | -0.279 | -0.139 | 0.008 | 0.302 | 1.006 | 990 | 292.6 | 1118.5 |
| Guangzhou | HRV | RSV_A | -0.217 | 0.202 | -0.591 | -0.358 | -0.226 | -0.079 | 0.190 | 1.005 | 1100 | 206.6 | 992.6 |
| Guangzhou | HPIV | RSV_B | 0.423 | 0.181 | 0.046 | 0.306 | 0.433 | 0.551 | 0.746 | 1.008 | 670 | 232.2 | 1204.7 |
| Guangzhou | HADV | RSV_B | 0.321 | 0.169 | -0.030 | 0.210 | 0.329 | 0.443 | 0.627 | 1.004 | 1300 | 238.2 | 1225.4 |
| Guangzhou | HMPV | RSV_B | 0.148 | 0.189 | -0.228 | 0.018 | 0.148 | 0.277 | 0.514 | 1.004 | 1400 | 246.7 | 1100.3 |
| Guangzhou | HCOV | RSV_B | 0.105 | 0.183 | -0.259 | -0.020 | 0.103 | 0.232 | 0.456 | 1.003 | 1700 | 242.4 | 1137.0 |
| Guangzhou | HBOV | RSV_B | 0.328 | 0.212 | -0.123 | 0.193 | 0.338 | 0.480 | 0.698 | 1.004 | 1700 | 270.9 | 1061.4 |
| Guangzhou | HRV | RSV_B | 0.701 | 0.136 | 0.381 | 0.621 | 0.720 | 0.802 | 0.903 | 1.005 | 1200 | 180.8 | 968.4 |
| Guangzhou | HADV | HPIV | -0.028 | 0.194 | -0.403 | -0.162 | -0.031 | 0.106 | 0.352 | 1.003 | 1800 | 252.2 | 1461.4 |
| Guangzhou | HMPV | HPIV | -0.041 | 0.188 | -0.397 | -0.171 | -0.044 | 0.086 | 0.337 | 1.004 | 1400 | 248.5 | 1322.4 |
| Guangzhou | HCOV | HPIV | 0.479 | 0.176 | 0.107 | 0.363 | 0.490 | 0.610 | 0.783 | 1.005 | 1200 | 225.8 | 1341.2 |
| Guangzhou | HBOV | HPIV | 0.262 | 0.237 | -0.219 | 0.098 | 0.270 | 0.434 | 0.698 | 1.007 | 820 | 277.6 | 1287.7 |
| Guangzhou | HRV | HPIV | 0.558 | 0.209 | 0.076 | 0.428 | 0.593 | 0.716 | 0.866 | 1.007 | 970 | 187.6 | 1082.9 |
| Guangzhou | HMPV | HADV | 0.095 | 0.176 | -0.254 | -0.027 | 0.097 | 0.218 | 0.433 | 1.003 | 2000 | 261.7 | 1348.5 |
| Guangzhou | HCOV | HADV | 0.114 | 0.181 | -0.231 | -0.015 | 0.115 | 0.245 | 0.463 | 1.001 | 4100 | 260.4 | 1390.6 |
| Guangzhou | HBOV | HADV | 0.308 | 0.219 | -0.129 | 0.154 | 0.314 | 0.467 | 0.712 | 1.004 | 1500 | 292.2 | 1316.8 |
| Guangzhou | HRV | HADV | -0.015 | 0.233 | -0.450 | -0.175 | -0.014 | 0.140 | 0.443 | 1.007 | 830 | 218.7 | 1127.0 |
| Guangzhou | HCOV | HMPV | -0.015 | 0.190 | -0.396 | -0.140 | -0.010 | 0.116 | 0.342 | 1.002 | 2400 | 251.0 | 1245.8 |
| Guangzhou | HBOV | HMPV | 0.526 | 0.202 | 0.075 | 0.411 | 0.549 | 0.673 | 0.837 | 1.011 | 580 | 277.0 | 1168.1 |
| Guangzhou | HRV | HMPV | 0.057 | 0.269 | -0.477 | -0.132 | 0.062 | 0.247 | 0.559 | 1.013 | 450 | 196.7 | 993.9 |
| Guangzhou | HBOV | HCOV | 0.147 | 0.223 | -0.304 | -0.005 | 0.158 | 0.307 | 0.554 | 1.004 | 1300 | 265.9 | 1196.7 |
| Guangzhou | HRV | HCOV | 0.125 | 0.241 | -0.350 | -0.040 | 0.127 | 0.293 | 0.592 | 1.001 | 5500 | 209.7 | 1041.6 |
| Guangzhou | HRV | HBOV | 0.659 | 0.167 | 0.262 | 0.560 | 0.691 | 0.785 | 0.895 | 1.007 | 790 | 196.5 | 939.4 |
| Chongqing | IBV | IAV | -0.022 | 0.221 | -0.424 | -0.178 | -0.032 | 0.122 | 0.424 | 1.014 | 410 | 214.5 | 827.4 |
| Chongqing | RSV_A | IAV | -0.094 | 0.132 | -0.340 | -0.185 | -0.096 | -0.007 | 0.177 | 1.002 | 3200 | 247.1 | 1190.2 |
| Chongqing | RSV_B | IAV | 0.330 | 0.130 | 0.062 | 0.243 | 0.335 | 0.423 | 0.564 | 1.002 | 3000 | 231.9 | 1156.8 |
| Chongqing | HPIV | IAV | -0.413 | 0.170 | -0.710 | -0.533 | -0.424 | -0.306 | -0.052 | 1.006 | 1100 | 276.5 | 1274.8 |
| Chongqing | HADV | IAV | -0.302 | 0.159 | -0.590 | -0.416 | -0.310 | -0.197 | 0.032 | 1.001 | 4600 | 228.7 | 1058.3 |
| Chongqing | HMPV | IAV | -0.716 | 0.169 | -0.928 | -0.842 | -0.752 | -0.627 | -0.313 | 1.011 | 530 | 196.1 | 874.3 |
| Chongqing | HCOV | IAV | -0.405 | 0.283 | -0.858 | -0.618 | -0.432 | -0.232 | 0.222 | 1.015 | 430 | 263.2 | 946.6 |
| Chongqing | HBOV | IAV | -0.417 | 0.143 | -0.671 | -0.522 | -0.426 | -0.323 | -0.111 | 1.004 | 1400 | 256.5 | 1136.2 |
| Chongqing | HRV | IAV | -0.515 | 0.174 | -0.808 | -0.641 | -0.527 | -0.405 | -0.134 | 1.008 | 720 | 204.0 | 947.1 |
| Chongqing | RSV_A | IBV | 0.436 | 0.177 | 0.085 | 0.313 | 0.437 | 0.565 | 0.768 | 1.008 | 940 | 232.6 | 1001.2 |
| Chongqing | RSV_B | IBV | 0.028 | 0.167 | -0.303 | -0.086 | 0.027 | 0.142 | 0.348 | 1.004 | 1500 | 216.3 | 966.6 |
| Chongqing | HPIV | IBV | 0.031 | 0.209 | -0.372 | -0.113 | 0.029 | 0.172 | 0.442 | 1.007 | 760 | 250.7 | 1074.5 |
| Chongqing | HADV | IBV | -0.381 | 0.194 | -0.724 | -0.519 | -0.393 | -0.255 | 0.021 | 1.008 | 770 | 212.2 | 869.6 |
| Chongqing | HMPV | IBV | -0.107 | 0.408 | -0.787 | -0.416 | -0.139 | 0.187 | 0.736 | 1.041 | 170 | 204.7 | 710.6 |
| Chongqing | HCOV | IBV | 0.469 | 0.317 | -0.320 | 0.288 | 0.519 | 0.716 | 0.890 | 1.028 | 220 | 239.0 | 747.1 |
| Chongqing | HBOV | IBV | -0.387 | 0.219 | -0.771 | -0.544 | -0.402 | -0.241 | 0.062 | 1.017 | 440 | 229.7 | 937.0 |
| Chongqing | HRV | IBV | -0.009 | 0.266 | -0.512 | -0.197 | -0.012 | 0.176 | 0.509 | 1.010 | 580 | 191.7 | 839.5 |
| Chongqing | RSV_B | RSV_A | -0.553 | 0.083 | -0.702 | -0.611 | -0.558 | -0.499 | -0.377 | 1.002 | 3700 | 255.5 | 1342.6 |
| Chongqing | HPIV | RSV_A | -0.027 | 0.128 | -0.264 | -0.118 | -0.030 | 0.057 | 0.232 | 1.003 | 2300 | 282.1 | 1435.8 |
| Chongqing | HADV | RSV_A | -0.232 | 0.117 | -0.447 | -0.314 | -0.236 | -0.157 | 0.008 | 1.001 | 5400 | 229.5 | 1213.1 |
| Chongqing | HMPV | RSV_A | 0.155 | 0.299 | -0.417 | -0.047 | 0.144 | 0.354 | 0.774 | 1.027 | 300 | 240.6 | 1078.0 |
| Chongqing | HCOV | RSV_A | 0.294 | 0.280 | -0.274 | 0.111 | 0.293 | 0.480 | 0.825 | 1.026 | 270 | 266.2 | 1105.4 |
| Chongqing | HBOV | RSV_A | 0.015 | 0.124 | -0.225 | -0.070 | 0.014 | 0.099 | 0.255 | 1.001 | 6000 | 247.8 | 1282.3 |
| Chongqing | HRV | RSV_A | -0.014 | 0.153 | -0.315 | -0.121 | -0.017 | 0.089 | 0.286 | 1.003 | 2200 | 215.2 | 1120.7 |
| Chongqing | HPIV | RSV_B | -0.180 | 0.137 | -0.445 | -0.274 | -0.180 | -0.089 | 0.091 | 1.002 | 2900 | 274.2 | 1409.8 |
| Chongqing | HADV | RSV_B | -0.115 | 0.140 | -0.384 | -0.213 | -0.117 | -0.019 | 0.158 | 1.002 | 2800 | 233.4 | 1201.3 |
| Chongqing | HMPV | RSV_B | -0.071 | 0.333 | -0.688 | -0.300 | -0.086 | 0.139 | 0.644 | 1.018 | 360 | 237.9 | 1057.0 |
| Chongqing | HCOV | RSV_B | -0.019 | 0.354 | -0.757 | -0.239 | -0.033 | 0.198 | 0.726 | 1.043 | 660 | 270.5 | 1094.6 |
| Chongqing | HBOV | RSV_B | -0.213 | 0.141 | -0.475 | -0.309 | -0.217 | -0.120 | 0.079 | 1.002 | 2400 | 241.5 | 1260.3 |
| Chongqing | HRV | RSV_B | -0.120 | 0.165 | -0.432 | -0.234 | -0.124 | -0.005 | 0.205 | 1.002 | 3600 | 210.7 | 1092.4 |
| Chongqing | HADV | HPIV | 0.250 | 0.172 | -0.102 | 0.136 | 0.256 | 0.372 | 0.571 | 1.006 | 940 | 272.5 | 1309.0 |
| Chongqing | HMPV | HPIV | 0.560 | 0.224 | 0.038 | 0.427 | 0.593 | 0.732 | 0.885 | 1.017 | 330 | 247.5 | 1136.8 |
| Chongqing | HCOV | HPIV | -0.213 | 0.346 | -0.762 | -0.462 | -0.253 | -0.019 | 0.625 | 1.027 | 280 | 285.9 | 1180.9 |
| Chongqing | HBOV | HPIV | 0.276 | 0.179 | -0.089 | 0.155 | 0.282 | 0.402 | 0.604 | 1.001 | 5400 | 290.1 | 1377.9 |
| Chongqing | HRV | HPIV | 0.488 | 0.157 | 0.156 | 0.387 | 0.497 | 0.602 | 0.763 | 1.005 | 1300 | 235.6 | 1173.6 |
| Chongqing | HMPV | HADV | 0.123 | 0.321 | -0.566 | -0.080 | 0.140 | 0.344 | 0.719 | 1.018 | 340 | 215.8 | 937.9 |
| Chongqing | HCOV | HADV | 0.149 | 0.346 | -0.497 | -0.101 | 0.139 | 0.405 | 0.796 | 1.028 | 220 | 258.4 | 984.8 |
| Chongqing | HBOV | HADV | 0.575 | 0.118 | 0.318 | 0.502 | 0.584 | 0.660 | 0.777 | 1.002 | 3300 | 227.7 | 1152.0 |
| Chongqing | HRV | HADV | 0.386 | 0.221 | -0.078 | 0.237 | 0.400 | 0.546 | 0.769 | 1.004 | 1600 | 204.6 | 1007.0 |
| Chongqing | HCOV | HMPV | 0.415 | 0.392 | -0.539 | 0.186 | 0.511 | 0.730 | 0.899 | 1.018 | 340 | 266.7 | 837.2 |
| Chongqing | HBOV | HMPV | -0.390 | 0.278 | -0.837 | -0.597 | -0.421 | -0.217 | 0.231 | 1.018 | 350 | 236.1 | 1008.8 |
| Chongqing | HRV | HMPV | 0.562 | 0.224 | 0.041 | 0.436 | 0.593 | 0.732 | 0.890 | 1.007 | 1000 | 179.9 | 874.4 |
| Chongqing | HBOV | HCOV | 0.249 | 0.311 | -0.371 | 0.038 | 0.250 | 0.474 | 0.804 | 1.026 | 260 | 273.3 | 1048.2 |
| Chongqing | HRV | HCOV | 0.007 | 0.415 | -0.748 | -0.311 | -0.004 | 0.322 | 0.775 | 1.025 | 310 | 204.2 | 900.9 |
| Chongqing | HRV | HBOV | 0.511 | 0.223 | 0.007 | 0.370 | 0.540 | 0.678 | 0.859 | 1.010 | 580 | 213.2 | 1051.6 |
| n.eff: effective sample size; DIC: Deviance Information Criterion; pD: effective number of parameters | | | | | | | | | | | | | |
